# Efficacy and safety of TACE combined with traditional Chinese medicine versus TACE alone in hepatocellular carcinoma: bayesian network meta-analysis and pharmacological mechanisms study

**DOI:** 10.3389/fphar.2024.1495343

**Published:** 2024-12-06

**Authors:** Li Chen, Xiu-Ling Zhu, Jie Lin, Dong-Liang Li

**Affiliations:** ^1^ Department of Hepatobiliary Disease, Fuzong Clinical Medical College of Fujian Medical University, Fuzhou, Fujian, China; ^2^ Department of Hepatobiliary Disease, 900 Hospital of the Joint Logistics Team of the Chinese PLA, Fuzhou, Fujian, China; ^3^ Department of Hepatobiliary and Pancreatic Surgery, Second Affiliated Hospital of Jilin University, Changchun, Jilin, China

**Keywords:** hepatocellular carcinoma, transarterial chemoembolization, traditional Chinese medicine, bayesian network meta-analysis, network pharmacology, molecular docking

## Abstract

**Purpose:**

This study investigates the clinical benefits of integrating traditional Chinese medicine (TCM) with Transarterial Chemoembolization (TACE) in hepatocellular carcinoma (HCC) treatment via meta-analysis and an exploration of network pharmacology analysis (NPA).

**Methods:**

A comprehensive search across different databases retrieved all randomized controlled trials (RCTs) evaluating TCM combined with TACE for HCC. Meta-analysis included 39 RCTs to assess the intervention effects. The bayesian network meta-analysis observed the relative efficacy and potential ranking of various interventions. Active compounds and target genes from frequently used TCM were sourced from the TCMSP database, while HCC disease targets were collected from five public disease databases. Regulatory networks connecting target genes with active components of key herbs were constructed. Following the identification of key genes, we conducted analyses of Gene Ontology (GO) and Kyoto Encyclopedia of Genes and Genomes (KEGG) to enrich our understanding of their functions. NPA and molecular docking methods were refined to reveal potential interactions between TCM components and their specific targets.

**Results:**

The combination of TCM with TACE significantly enhances the efficacy and safety of HCC treatment, improving the overall response rate, disease control rate, and overall survival rate, while also reducing the incidence of adverse events. Among the TCM evaluated, Ganfu Formula proved to be the most effective in enhancing patient response rates. Analysis of all included medicinal herbs identified 10 pivotal TCMs and 17 core genes. GO analysis revealed their significance in protein interactions, whereas KEGG analysis highlighted their role in crucial oncological pathways. NPA and molecular docking techniques elucidate the underlying mechanisms of action of TCM components.

**Conclusion:**

Adding TCM to TACE protocols significantly enhances treatment outcomes and safety in HCC patients by modulating tumor biology and systemic immune responses, highlighting its potential as an effective adjunct therapy. These findings support the inclusion of TCM in standard care regimens, offering potential for improved management of HCC.

**Systematic Review Registration:**

https://www.crd.york.ac.uk/PROSPERO/, identifier CRD42024571280.

## 1 Introduction

Hepatocellular carcinoma (HCC) ranks among the top three most common causes of cancer-associated mortality globally (Bray et al., 2024). Transarterial Chemoembolization (TACE) is a commonly employed therapeutic approach for HCC, especially in patients who are in the intermediate to advanced stages of the disease (Chang et al., 2020). However, TACE alone presents several limitations, including incomplete local treatment, absence of systemic effects and increased risks of tumor recurrence and metastasis (Kudo et al., 2020). Although TACE can deliver high concentrations of cytotoxic agents to tumor cells locally, it cannot effectively inhibit angiogenesis or disrupt the complex signaling networks within the tumor microenvironment, resulting in suboptimal therapeutic outcomes and diminished patient quality of life (Kudo, 2019).

Recently, the integration of traditional Chinese medicine (TCM) into therapeutic strategies for HCC has attracted heightened focus. TCM can effectively overcome the limitations of TACE through multi-targeted and multi-pathway mechanisms. Specific TCM components can inhibit angiogenesis, reducing the risks of tumor recurrence and metastasis (Li et al., 2022). Additionally, by modulating the complex signaling networks within the tumor microenvironment, TCM can enhance the systemic therapeutic effects of TACE, further improving overall efficacy and patient quality of life (Liao et al., 2020).

To rigorously assess the combined effectiveness of TCM and TACE in managing HCC, this study proposes to use meta- and network meta-analysis methods to integrate existing clinical trial data. Additionally, applying network pharmacology analysis (NPA) alongside molecular docking methods will help elucidate the underlying biological mechanisms of TCM. Bayesian network meta-analysis enables comparison of different treatment regimens’ effects, and offers more comprehensive efficacy assessments through indirect comparisons (Ahn and Kang, 2021). Incorporating NPA with molecular docking techniques (Zhao et al., 2023) uncovers how TCM components interact with their biological targets, providing theoretical support for clinical applications. This research aims to generate solid clinical evidence supporting the concurrent application of TCM with TACE for treating HCC, serving as a foundation for future scientific exploration and pharmaceutical innovation.

## 2 Materials and methods

### 2.1 Meta-analysis method of TCM for HCC

This study employs a network meta-analysis method as stipulated by the PRISMA guidelines. Project details have been recorded and can be found on the PROSPERO database (Moher et al., 2015) under the identifier CRD42024571280.

### 2.2 Database search strategy

Searches were conducted across both English-language and Chinese-language databases including PubMed, Embase, Web of Science, Cochrane Library, Chinese National Knowledge Infrastructure (CNKI), Chinese Biomedical Literature Database (CBM), China Science and Technology Journal Database (VIP), and Wanfang Data to retrieve records available up to 5 July 2024, without imposing language restrictions. Key search terms included “Hepatocellular Carcinoma,” “Traditional Chinese Medicine,” “randomized controlled trials.” Supplementary Table S1 includes the complete search methodologies.

### 2.3 Inclusion and exclusion criteria

Inclusion criteria: (1) All clinical randomized controlled trials (RCTs) assessing the effectiveness of combining TCM with TACE for treating HCC. (2) HCC diagnosis confirmed by pathological examination or established diagnostic criteria (Cannella, Zins, and Brancatelli, 2024). (3) Experimental group (EG) receiving TCM combined with TACE, control group (CG) receiving TACE treatment alone. (4) Defined outcome measures. Exclusion criteria: (1) Non-RCTs. (2) Studies unrelated to HCC. (3) Animal studies. (4) Reviews, meta-analyses, conference proceedings, letters.

### 2.4 Data extraction and quality assessment

LC and X-LZ meticulously screened and synthesized data, adhering strictly to predefined inclusion and exclusion criteria, in line with the Cochrane Collaboration’s assessment framework (Cumpston et al., 2019). Extracted information included the primary author’s name, sample size, the year of publication, participant demographics, details of the interventions, gender composition, age and outcome indicators including overall survival (OS), overall response rate (ORR), disease control rate (DCR) and adverse events (AEs). Any discrepancies were resolved by verifying the original texts or through third-party adjudication.

### 2.5 Statistical analysis

The data analysis utilized Review Manager 5.2 alongside STATA 12, focusing on odds ratios (OR) to measure effect sizes for binary outcomes. When both *p* ≥ 0.1 and I^2^ ≤ 50% suggested minimal heterogeneity, a fixed-effects model was employed. Conversely, significant heterogeneity prompted an exploration of underlying causes and the employment of a random-effects model. Sensitivity analysis assessed the heterogeneity sources and result robustness. Egger’s and Begg’s tests were utilized to assess the potential existence of publication bias. Network meta-analysis was conducted using Bayesian methods with the GeMTC package in R 4.1.0. Model convergence was assessed using residual plots. This analytical framework helped to compare the impacts across study groups, enhancing our understanding of different interventions’ effectiveness.

### 2.6 Exploration and targeting of TCM

The top 10 most frequently used TCM prescriptions extracted from the meta-analysis studies were selected as the main subjects for study. The bioactive components and corresponding drug targets of these herbs were acquired from the Traditional Chinese Medicine Systems Pharmacology Database and Analysis Platform (TCMSP, https://tcmspw.com/index.php) (Ru et al., 2014). The selection criteria were set with an oral bioavailability threshold of 30% or higher and a drug-likeness index of at least 0.18. Subsequent gene annotation of these component targets was conducted using human gene information downloaded from The Universal Protein Database (UniProt, https://legacy.uniprot.org/) (Bairoch et al., 2005). To compile HCC-specific disease targets, we accessed several databases: GeneCards (https://www.genecards.org/), Therapeutic Target Database (TTD, http://db.idrblab.net/ttd/), DisGeNet (https://www.disgenet.org/), Online Mendelian Inheritance in Man (OMIM, http://omim.org/), and Comparative Toxicogenomics Database (CTD, https://ctdbase.org/). Gene intersections between TCM and HCC were identified using the ‘VennDiagram’ tool. The intersecting genes were then submitted to the STRING database (https://stringdb.org/), specifying humans as the target species, with a confidence score threshold set at > 0.4 to ensure interaction reliability. Independent proteins were excluded from the network to focus on biologically meaningful interactions. The resulting data were then imported into Cytoscape 3.8.2 (Doncheva et al., 2019) to generate a molecular network diagram of TCM components and intersecting genes. Central genes were identified using the Cytoscape software plugin cytoHubba, employing algorithms including Maximal Clique Centrality (MCC), Maximum Neighborhood Component (MNC), Degree, Closeness, Radiality, Stress, and Edge Percolated Component (EPC), which facilitated the selection of pivotal genes.

### 2.7 Network pharmacology analysis grounded in TCM hubs

Functional enrichment analysis, incorporating Gene Ontology (GO) and Kyoto Encyclopedia of Genes and Genomes (KEGG) pathway evaluations, was conducted to elucidate interaction pathways between TCM components and HCC-related genes. Essential genes were analyzed using packages like clusterProfiler and enrichplot, with *p*-value and q-value thresholds set at < 0.05. The UniProt database was used to retrieve the protein IDs for hub targets, and the corresponding 3D structures of each target protein were downloaded from the PDB database (https://www.rcsb.org/) (Shin and Cho, 2005). To minimize interference from non-essential molecules in subsequent molecular docking, water molecules, cofactors, and heteroatoms were removed using PyMOL software (Seeliger and de Groot, 2010), retaining only the core protein structure. Hydrogen atoms were then added to the protein, and energy minimization was conducted with AutoDockTools (Goodsell et al., 2021) to optimize the structure by removing geometric conflicts and relieving conformational strain, ensuring stability. Small molecule ligands’ 2D chemical structures from PubChem (Wang et al., 2012) (https://pubchem.ncbi.nlm.nih.gov/) were converted into 3D models by the application of ChemBio3D Ultra 14.0, with subsequent calculations performed to optimize the structures. Potential binding sites on the proteins were identified using AutoDockTools, and docking simulations were carried out with AutoDock Vina to determine the optimal binding configurations, visualized through PyMOL.

### 2.8 *In vitro* validation of differential gene expression

To verify core gene expression in HCC, we obtained the TCGA-LIHC dataset from the Genomic Data Commons (GDC, https://gdc.cancer.gov/). Then we acquired the HCC cell line HepG2 (CL-0103) and the human liver epithelial cell line THLE-2 (CL-0833) from Punose Biotechnology Co., Ltd. (Wuhan, China). Both cell lines were cultured in DMEM basal medium (Meilunbio, MA0212), supplemented with 10% fetal bovine serum (Opcel, BS-1105) and 1% penicillin/streptomycin (Meilunbio, MA0110). At 70%–90% confluence, total RNA was extracted using the TransZol Up Plus RNA Kit (Transgene Biotech, ER501-01-V2, China). Genomic DNA was removed with gDNA Purge (Novoprotein, E047-01A, China) according to the manufacturer’s instructions, and cDNA was synthesized. Quantitative reverse transcription PCR (qRT-PCR) was then performed using NovoStart^®^ SYBR qPCR SuperMix Plus (Novoprotein, E09601B, China) on an Applied Biosystems 7,300 Real-Time PCR system (Applied Biosystems, United States) to assess mRNA expression levels of core genes identified as stable binding targets in molecular docking analyses. qRT-PCR primers were synthesized by SunYa (Fuzhou, China), with primer sequences provided in the Supplementary Table S2. qRT-PCR cycling conditions were 95°C for 30 s for pre-denaturation, followed by 40 cycles at 95°C for 5 s and 60°C for 15 s for annealing. qRT-PCR data were analyzed using the 2^−ΔΔCT^ method.

## 3 Results

### 3.1 Literature retrieval and screening

**FIGURE 1:**
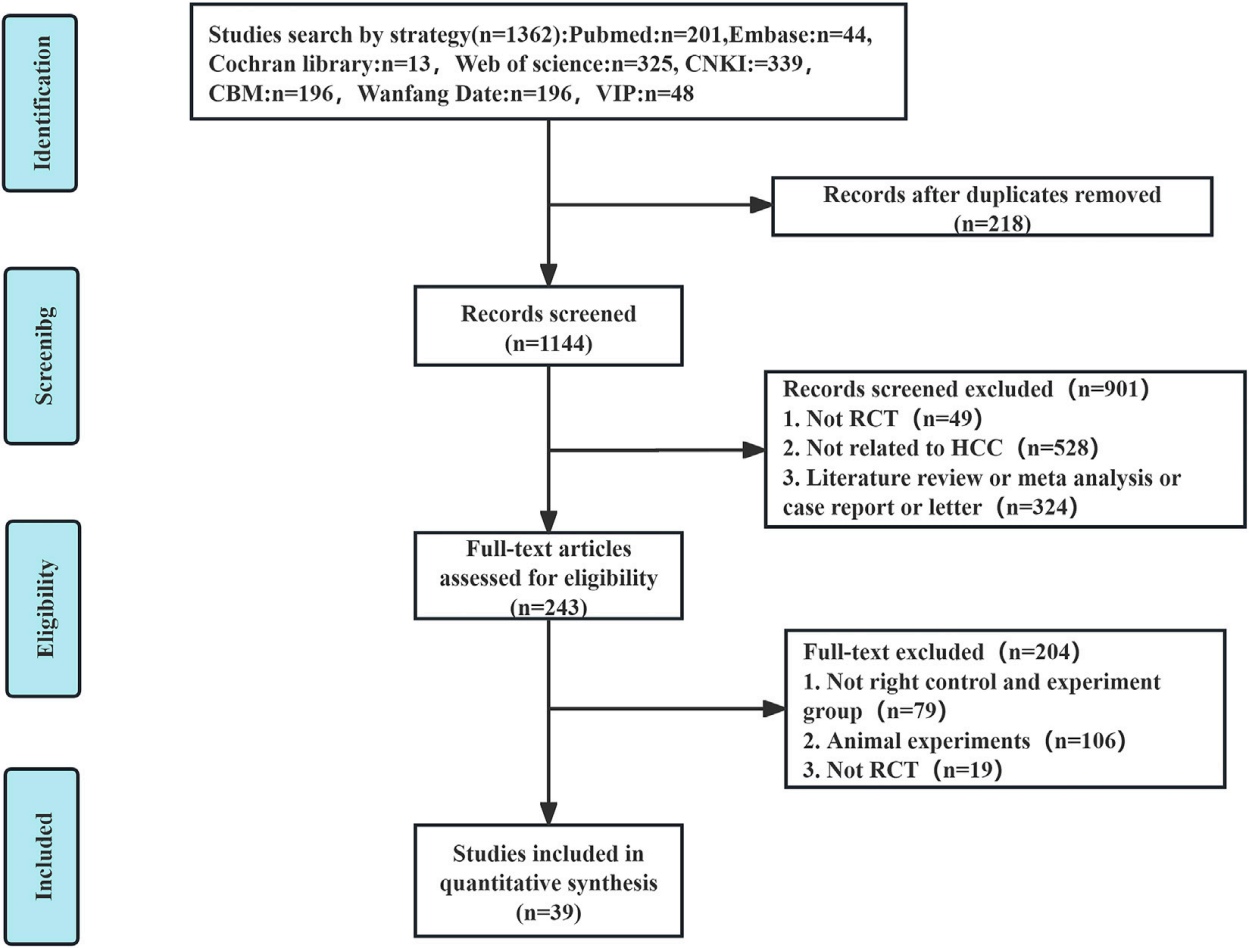
The flowchart for screening literature.

An extensive database search identified 1,362 relevant articles from sources including PubMed, Embase, Cochrane Library, Web of Science, CNKI, CBM, Wanfang Data, and VIP. Following the removal of 218 duplicate entries, an additional 901 articles were excluded after preliminary assessments. Of the 243 remaining articles, detailed evaluations excluded 79 for non-compliance with group criteria, 106 for being animal studies, and 19 for lacking randomization, resulting in 39 (Zhou et al., 2002; Shao et al., 2001; Ren and Cheng, 2004; Cao et al., 2005; Zhang C. Q. et al., 2005; Zhang Y. M. et al., 2005; Lin and Guo, 2005; Yuan et al., 2005; Ou et al., 2006; Wen and Peng, 2006; Yu et al., 2006; Liu et al., 2007; Wang and Chen, 2007; Zhang et al., 2007; Zhong et al., 2007; Wu et al., 2010; Zhou et al., 2010; Han et al., 2013; Liu et al., 2013; Song et al., 2013; Zhao et al., 2013; Yin et al., 2013; Li et al., 2014; Zheng, 2014; Li et al., 2015; Tang, 2017; Ding, 2018; Wan, 2018; Yang, 2018; Huang, 2019; Li et al., 2019; Qiao J., 2019; Tian et al., 2019; Qiao X. et al., 2019; Hong et al., 2020; Wang et al., 2020; Meng et al., 2021; Yan et al., 2021; Tong et al., 2022) eligible RCTs (Figure 1).

### 3.2 Basic characteristics of selected literature

The study included 3,939 HCC patients, with 2,027 assigned to the EG and 1,912 to the CG, the latter receiving only TACE therapy. The EG was administered a treatment regimen that combined TCM with TACE. The studies were divided based on TCM formulations into three categories: Liver-soothing (Shugan) herbs, Supporting vital qi (Fuzheng) herbs, and resolving blood stasis (Huayu) herbs, comprising 8 (Zhang C. Q. et al., 2005; Zhang Y. M. et al., 2005; Ou et al., 2006; Liu et al., 2013; Yang, 2018; Tian et al., 2019; Meng et al., 2021; Yan et al., 2021), 20 (Zhou et al., 2002; Ren and Cheng, 2004; Cao et al., 2005; Lin and Guo, 2005; Yuan et al., 2005; Wen and Peng, 2006; Wang and Chen, 2007; Han et al., 2013; Song et al., 2013; Zhao et al., 2013; Yin et al., 2013; Zheng, 2014; Li et al., 2015; Tang, 2017; Ding, 2018; Wan, 2018; Qiao J., 2019; Hong et al., 2020; Wang et al., 2020; Tong et al., 2022), and 11 (Shao et al., 2001; Yu et al., 2006; Liu et al., 2007; Zhong et al., 2007; Zhang et al., 2007; Wu et al., 2010; Zhou et al., 2010; Li et al., 2014; Huang, 2019; Li et al., 2019; Qiao X. et al., 2019) studies, respectively (Supplementary Table S3).

### 3.3 Quality assessment of included literature

**FIGURE 2:**
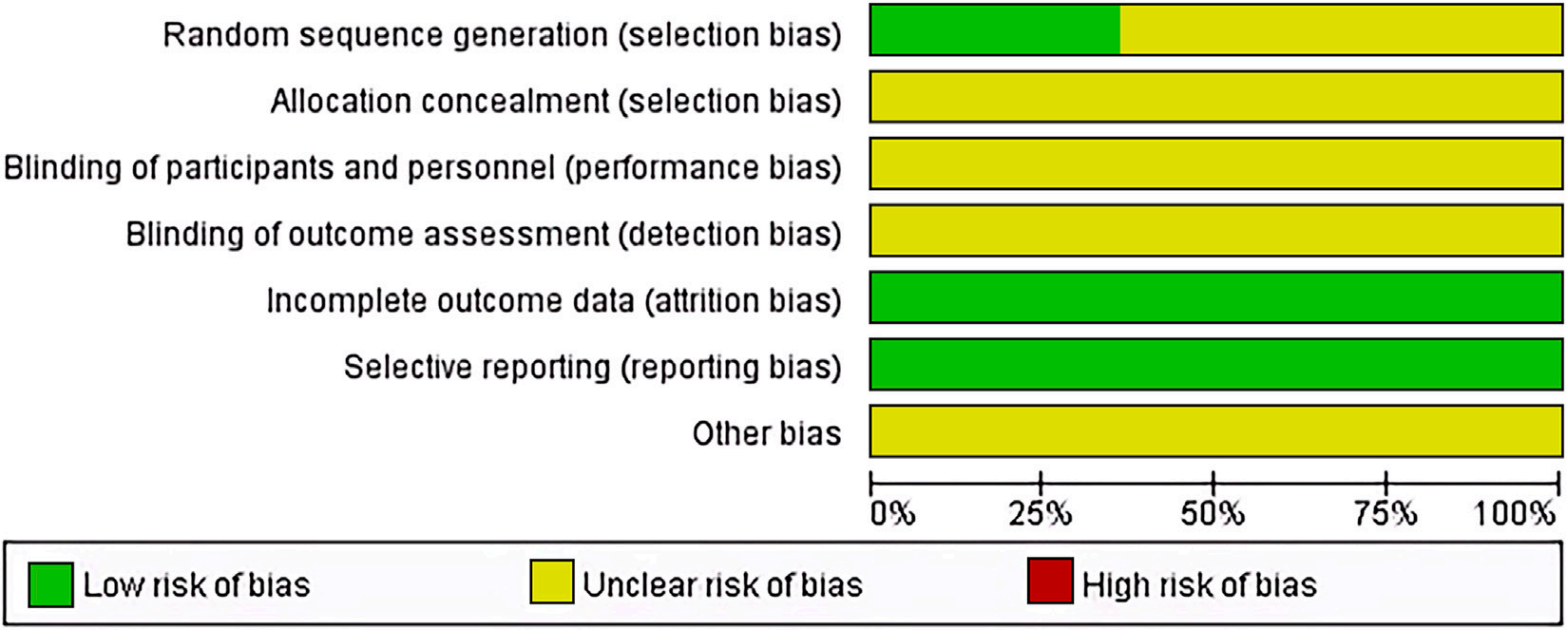
Risk assessment for the included studies.

14 articles (Shao et al., 2001; Ren and Cheng, 2004; Lin and Guo, 2005; Zhang et al., 2007; Wu et al., 2010; Zhao et al., 2013; Tang, 2017; Wan, 2018; Yang, 2018; Li et al., 2019; Qiao J., 2019; Tian et al., 2019; Yan et al., 2021; Tong et al., 2022) used random number generation methods and were considered low risk; the rest employed random grouping methods. Allocation concealment and blinding were not reported in any of the articles, reflecting the practical challenges of implementing these in non-drug therapies like TACE. All studies completed data collection and reporting, thus considered low risk. Unreported potential biases were noted as presenting an unclear risk of bias (Figure 2).

### 3.4 Clinical outcomes

#### 3.4.1 ORR and DCR

**FIGURE 3:**
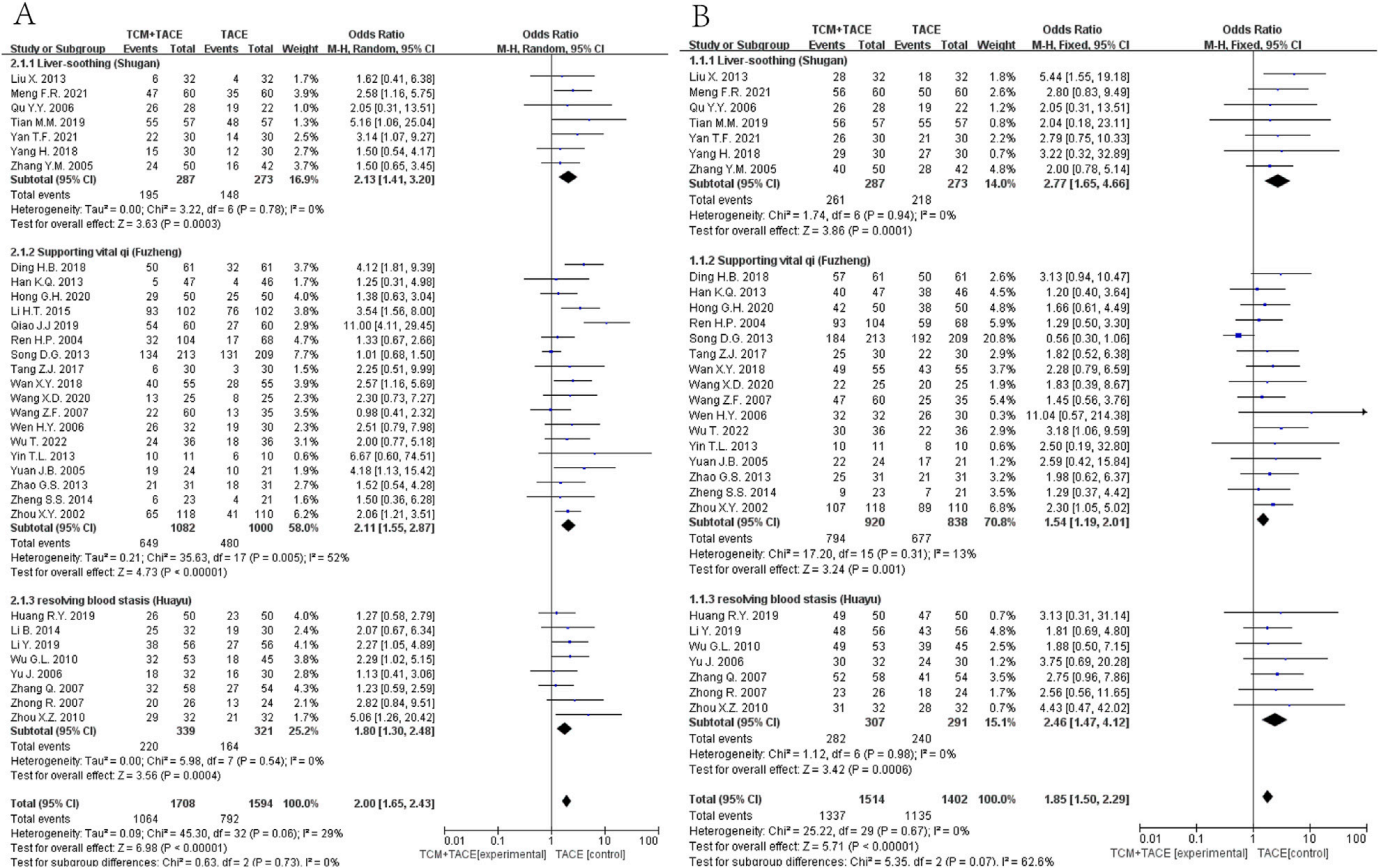
**(A)** ORR between EG and CG. **(B)** DCR between EG and CG.

Data from 33 (Zhang Y. M. et al., 2005; Ou et al., 2006; Liu et al., 2013; Yang, 2018; Tian et al., 2019; Meng et al., 2021; Yan et al., 2021; Zhou et al., 2002; Ren and Cheng, 2004; Yuan et al., 2005; Wen and Peng, 2006; Wang and Chen, 2007; Han et al., 2013; Song et al., 2013; Zhao et al., 2013; Yin et al., 2013; Zheng, 2014; Li et al., 2015; Tang, 2017; Ding, 2018; Wan, 2018; Qiao J., 2019; Hong et al., 2020; Wang et al., 2020; Tong et al., 2022; Yu et al., 2006; Zhong et al., 2007; Zhang et al., 2007; Wu et al., 2010; Zhou et al., 2010; Li et al., 2014; Huang, 2019; Li et al., 2019) and 30 studies (Zhang Y. M. et al., 2005; Ou et al., 2006; Liu et al., 2013; Yang, 2018; Tian et al., 2019; Meng et al., 2021; Yan et al., 2021; Zhou et al., 2002; Ren and Cheng, 2004; Yuan et al., 2005; Wen and Peng, 2006; Wang and Chen, 2007; Han et al., 2013; Song et al., 2013; Zhao et al., 2013; Yin et al., 2013; Zheng, 2014; Tang, 2017; Ding, 2018; Wan, 2018; Hong et al., 2020; Wang et al., 2020; Tong et al., 2022; Yu et al., 2006; Zhong et al., 2007; Zhang et al., 2007; Wu et al., 2010; Zhou et al., 2010; Huang, 2019; Li et al., 2019), respectively, indicated significant improvements in ORR and DCR when combining TCM with TACE versus TACE alone (OR = 2.00, 95% CI [1.65, 2.43], *p* < 0.00001; OR = 1.85, 95% CI [1.50, 2.29], *p* < 0.00001). Subgroup analyses also demonstrated that Liver-soothing (Shugan), Supporting vital qi (Fuzheng) herbs, and resolving blood stasis (Huayu) herbs each notably enhanced ORR and DCR in HCC patients (all *p*-values < 0.05) (Figure 3).

#### 3.4.2 OS

**FIGURE 4:**
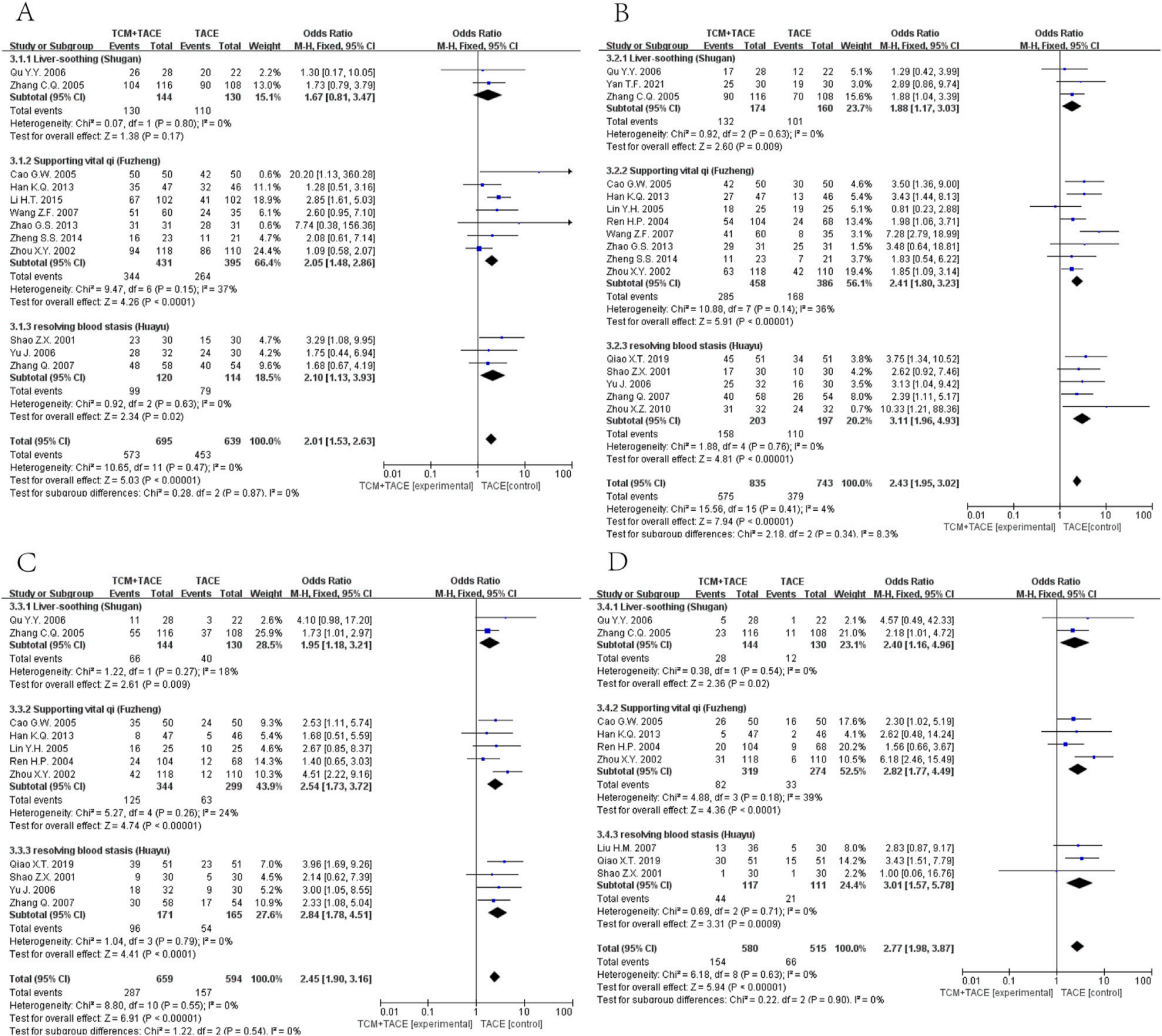
Forest plot and subgroup analysis. **(A)** half-year OS. **(B)** one-year OS. **(C)** two-year OS. **(D)** three-year OS.

Data on half-year, 1-year, 2-year, and 3-year OS were reported in 12 (Zhang C. Q. et al., 2005; Ou et al., 2006; Zhou et al., 2002; Cao et al., 2005; Wang and Chen, 2007; Han et al., 2013; Zhao et al., 2013; Zheng, 2014; Li et al., 2015; Shao et al., 2001; Yu et al., 2006; Zhang et al., 2007), 16 (Zhang C. Q. et al., 2005; Ou et al., 2006; Yan et al., 2021; Zhou et al., 2002; Ren and Cheng, 2004; Cao et al., 2005; Lin and Guo, 2005; Wang and Chen, 2007; Han et al., 2013; Zhao et al., 2013; Zheng, 2014; Shao et al., 2001; Yu et al., 2006; Zhang et al., 2007; Zhou et al., 2010; Qiao X. et al., 2019), 11 (Zhang C. Q. et al., 2005; Ou et al., 2006; Zhou et al., 2002; Ren and Cheng, 2004; Cao et al., 2005; Lin and Guo, 2005; Han et al., 2013; Shao et al., 2001; Yu et al., 2006; Zhang et al., 2007; Qiao X. et al., 2019) and 9 (Zhang C. Q. et al., 2005; Ou et al., 2006; Zhou et al., 2002; Ren and Cheng, 2004; Cao et al., 2005; Lin and Guo, 2005; Han et al., 2013; Shao et al., 2001; Yu et al., 2006; Zhang et al., 2007; Qiao X. et al., 2019) articles respectively. The meta-analysis revealed that the integration of TCM with TACE remarkably enhanced OS at all time points mentioned [OR = 2.01, 95% CI (1.53, 2.63), *p* < 0.00001; OR = 2.43, 95% CI (1.95, 3.02), *p* < 0.00001; OR = 2.45, 95% CI (1.90, 3.16), *p* < 0.00001; OR = 2.77, 95% CI (1.98, 3.87), *p* < 0.00001] (Figure 4).

#### 3.4.3 AEs

**FIGURE 5:**
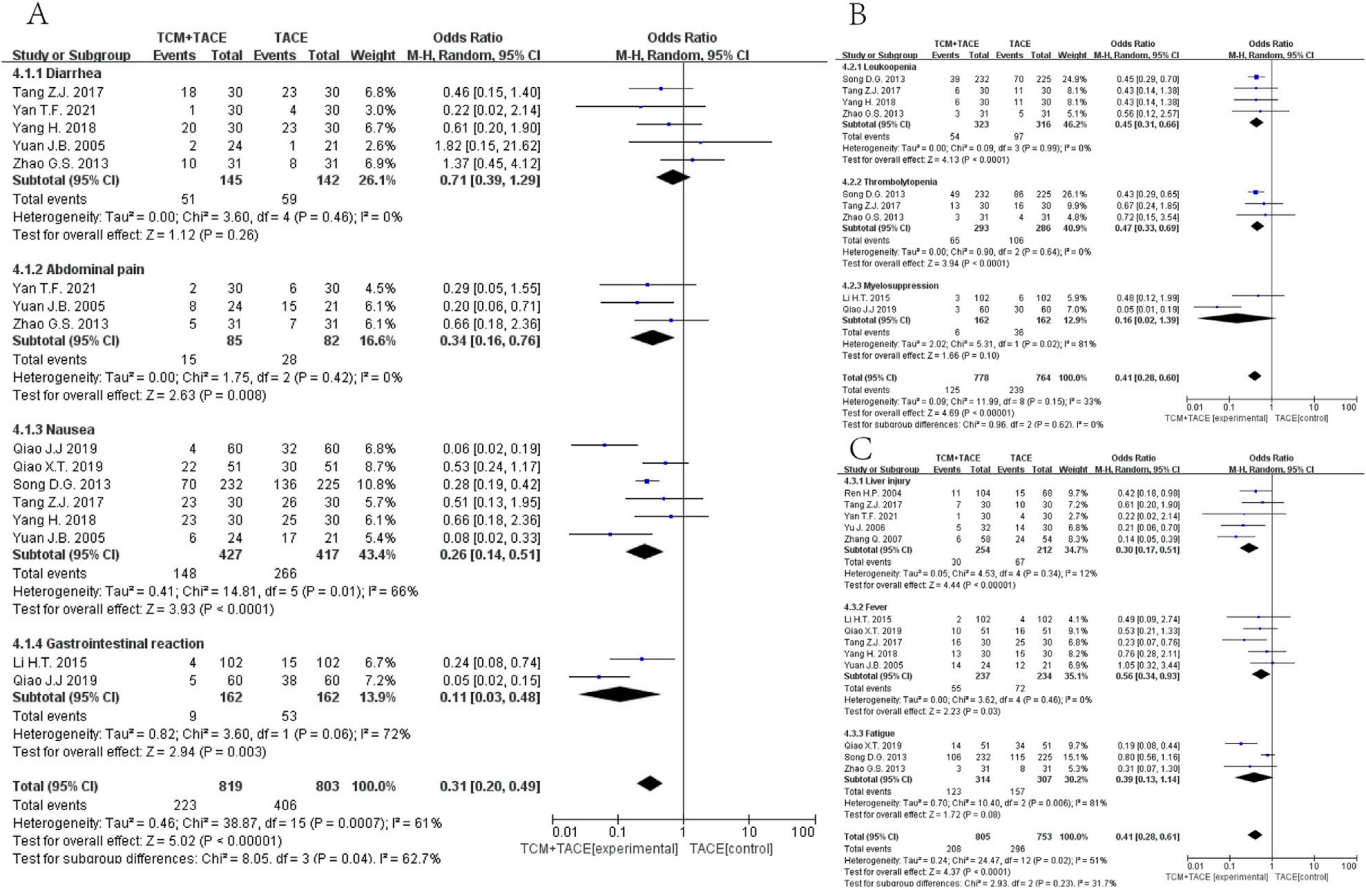
Forest plot and subgroup analysis. **(A)** Digestive system complications. **(B)** Hematologic system complications. **(C)** Other complications.

Nine articles (Yuan et al., 2005; Zhao et al., 2013; Tang, 2017; Yang, 2018; Yan et al., 2021; Song et al., 2013; Qiao J., 2019; Qiao X. et al., 2019; Li et al., 2015) (Figure 5A) reported on digestive system complications, showing that the EG had reduced incidences of abdominal pain [OR = 0.34, 95% CI (0.16, 0.76), *p* = 0.008], nausea [OR = 0.26, 95% CI (0.14, 0.51), *p* < 0.0001], and gastrointestinal reactions [OR = 0.11, 95% CI (0.03, 0.48), *p* = 0.003], with no obvious effect on diarrhea rates [OR = 0.71, 95% CI (0.39, 1.29), *p* = 0.26]. Six articles (Song et al., 2013; Zhao et al., 2013; Tang, 2017; Yang, 2018; Li et al., 2015; Qiao J., 2019) (Figure 5B) reported hematologic complications, where combined TCM treatment reduced occurrences of leukopenia [OR = 0.45, 95% CI (0.31, 0.66), *p* < 0.0001] and thrombocytopenia [OR = 0.47, 95% CI (0.33, 0.69), *p* < 0.0001], but did not significantly impact rates of myelosuppression [OR = 0.16, 95% CI (0.02, 1.39), *p* = 0.10]. For the remaining AEs, nine articles (Ren and Cheng, 2004; Yu et al., 2006; Zhang et al., 2007; Tang, 2017; Yan et al., 2021; Yuan et al., 2005; Li et al., 2015; Yang, 2018; Qiao X. et al., 2019; Song et al., 2013; Zhao et al., 2013) (Figure 5C) summarized that the EG improved occurrences of liver injury [OR = 0.30, 95% CI (0.17, 0.51), *p* < 0.00001] and fever [OR = 0.56, 95% CI (0.34, 0.93), *p* = 0.03] in HCC patients.

#### 3.4.4 Sensitivity analysis and publication bias

The sensitivity analysis demonstrated revealed that excluding any individual study did not alter the overall trend of the results, suggesting good stability of the study outcomes. Begg’s and Egger’s tests indicated the presence of publication bias for ORR (*p* = 0.049, *p* = 0.006). Further use of the trim-and-fill method, employing an iterative technique, estimated that 12 studies were missing; including these missing studies, the combined effect size estimate was 0.407 (*p* < 0.001), not substantially different from the original effect value of 0.372 (*p* < 0.001), suggesting minimal impact of publication bias and robust results. Detailed results are depicted in Supplementary Figures S1, S2.

#### 3.4.5 Network meta-analysis of drugs

**FIGURE 6:**
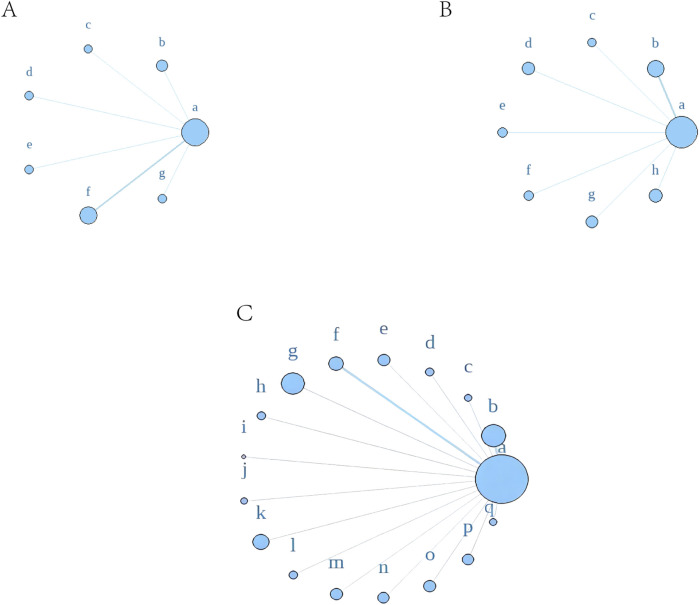
A network meta-analysis on ORR. **(A)** Liver-soothing (Shugan) herbs. **(B)** Resolving blood stasis (Huayu) herbs. **(C)** Supporting vital qi (Fuzheng) herbs. Notes: in **(A)** a: TACE, b: Shanxian Granule + TACE, c: E Tao Soup + TACE, d: Weitiao Formula II + TACE, e: Xiaoyao Powder + TACE, f: Xiao Chaihu Soup + TACE, g: Chaihu Shugan Powder + TACE. in **(B)** a: TACE, b: Guben Yiliu Formula II + TACE, c: Yiqi Huoxue Soup + TACE, d: Jinlong Capsule + TACE, e: Anti-cancer Formula + TACE, f: Jianpi Xiaotan Sanjie Formula + TACE, g: Ge Xia Zhu Yu Soup + TACE, h: Huangqin Soup + TACE. in **(C)** a: TACE, b: Jianpi Liqi Principle + TACE, c: Xiao Zheng Fuzheng Soup + TACE, d: Fuzheng Anti-cancer Formula + TACE, e: Fuzheng Pinggan Xiaoliu Soup + TACE, f: Fuzheng Jiedu Formula + TACE, g: Yangzheng Xiaoji Capsule + TACE, h: Huai Er Granule + TACE, i: Anti-cancer Formula + TACE, j: Songyou Drink + TACE, k: Jianpi Huayu Formula + TACE, l: Weitiao Formula II + TACE, m: Compound Hongdoushan Capsule + TACE, n: Zhengan Huazheng Formula + TACE, o: Ganfu Formula + TACE, p: Compound Banmao Capsule + TACE, q: Fuzheng Quxie Anti-cancer Formula + TACE.

**FIGURE 7:**
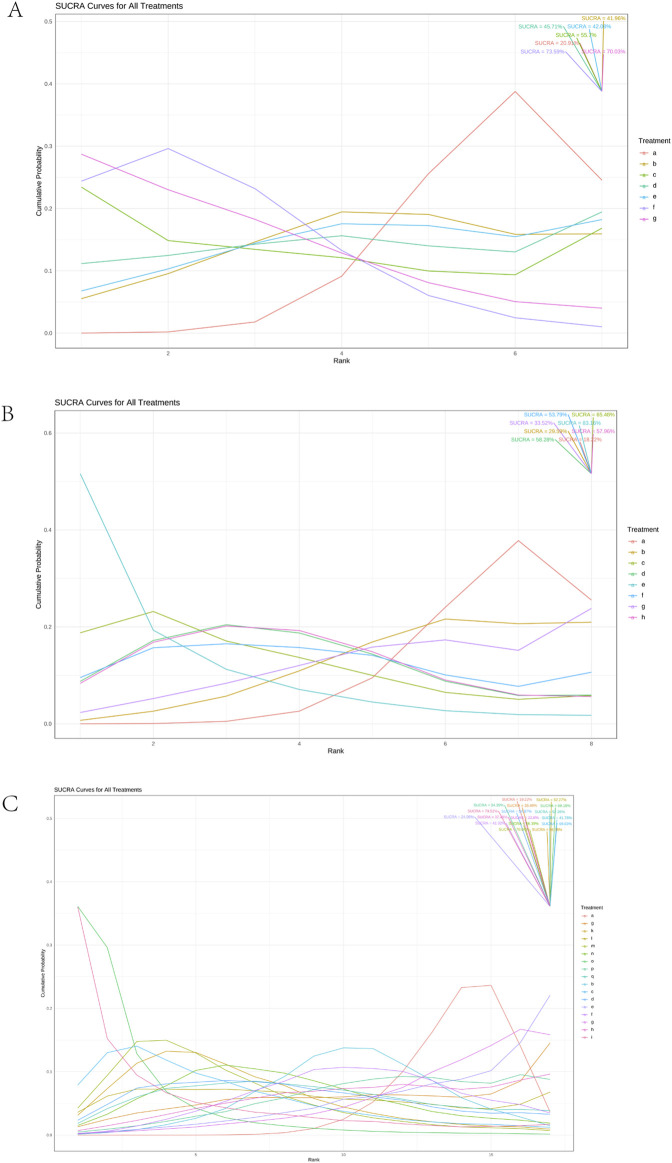
Results of SUCRA. **(A)** Liver-soothing (Shugan) herbs. **(B)** Resolving blood stasis (Huayu) herbs. **(C)** Supporting vital qi (Fuzheng) herbs. Notes: in **(A)** a: TACE, b: Shanxian Granule + TACE, c: E Tao Soup + TACE, d: Weitiao Formula II + TACE, e: Xiaoyao Powder + TACE, f: Xiao Chaihu Soup + TACE, g: Chaihu Shugan Powder + TACE. in **(B)** a: TACE, b: Guben Yiliu Formula II + TACE, c: Yiqi Huoxue Soup + TACE, d: Jinlong Capsule + TACE, e: Anti-cancer Formula + TACE, f: Jianpi Xiaotan Sanjie Formula + TACE, g: Ge Xia Zhu Yu Soup + TACE, h: Huangqin Soup + TACE. in **(C)** a: TACE, b: Jianpi Liqi Principle + TACE, c: Xiao Zheng Fuzheng Soup + TACE, d: Fuzheng Anti-cancer Formula + TACE, e: Fuzheng Pinggan Xiaoliu Soup + TACE, f: Fuzheng Jiedu Formula + TACE, g: Yangzheng Xiaoji Capsule + TACE, h: Huai Er Granule + TACE, i: Anti-cancer Formula + TACE, j: Songyou Drink + TACE, k: Jianpi Huayu Formula + TACE, l: Weitiao Formula II + TACE, m: Compound Hongdoushan Capsule + TACE, n: Zhengan Huazheng Formula + TACE, o: Ganfu Formula + TACE, p: Compound Banmao Capsule + TACE, q: Fuzheng Quxie Anti-cancer Formula + TACE.

Using a Bayesian network, the effects of liver-soothing (Shugan), resolving blood stasis (Huayu), and supporting vital qi (Fuzheng) herbs on ORR in EG and CG were compared (Figure 6). The leverage plot (Supplementary Figure S3) shows that the studies are distributed within the curve, indicating good convergence of the Bayesian model. The surface under the cumulative ranking curve (SUCRA) values, suggesting intervention effectiveness, favored treatments with higher scores for better ORR outcomes. For liver-soothing (Shugan) herbs, the top three ORR rankings were Xiao Chaihu Soup (73.59%), Chaihu Shugan Powder (70.03%), E Tao Soup (55.70%) (Figure 7A); for resolving blood stasis (Huayu) herbs, the top three were Anti-cancer Formula (83.16%), Yiqi Huoxue Soup (65.48%), Jinlong Capsule (58.28%) (Figure 7B); and for supporting vital qi (Fuzheng) herbs, the top three were Ganfu Formula (89.19%), Anti-Cancer Formula (79.52%), Compound Hongdoushan Capsule (70.04%) (Figure 7C).

**FIGURE 8:**
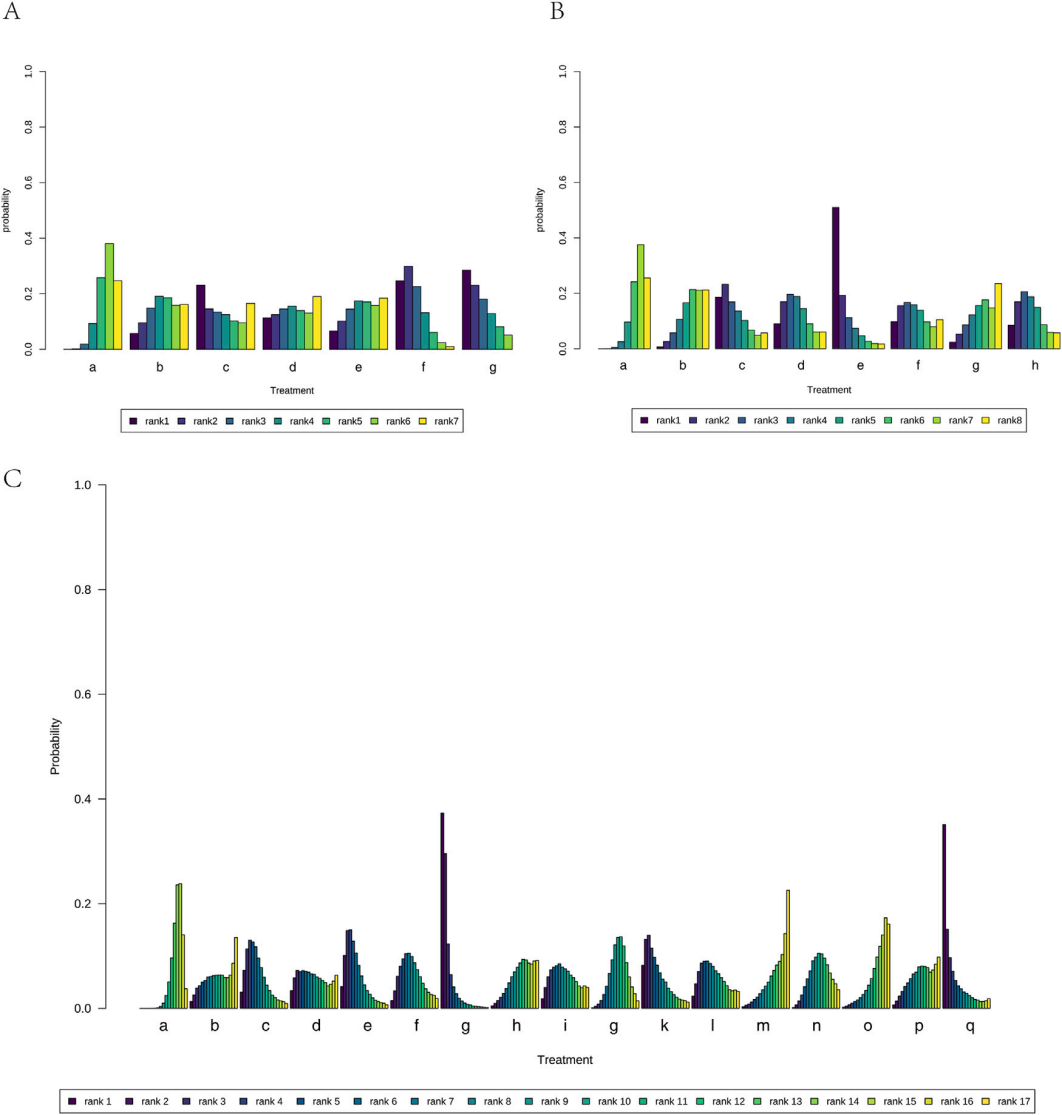
Ranking probability results for all treatment groups under different possible rankings. **(A)** Liver-soothing (Shugan) herbs. **(B)** Resolving blood stasis (Huayu) herbs. **(C)** Supporting vital qi (Fuzheng) herbs. Notes: in **(A)** a: TACE, b: Shanxian Granule + TACE, c: E Tao Soup + TACE, d: Weitiao Formula II + TACE, e: Xiaoyao Powder + TACE, f: Xiao Chaihu Soup + TACE, g: Chaihu Shugan Powder + TACE. in **(B)** a: TACE, b: Guben Yiliu Formula II + TACE, c: Yiqi Huoxue Soup + TACE, d: Jinlong Capsule + TACE, e: Anti-cancer Formula + TACE, f: Jianpi Xiaotan Sanjie Formula + TACE, g: Ge Xia Zhu Yu Soup + TACE, h: Huangqin Soup + TACE. in **(C)** a: TACE, b: Jianpi Liqi Principle + TACE, c: Xiao Zheng Fuzheng Soup + TACE, d: Fuzheng Anti-cancer Formula + TACE, e: Fuzheng Pinggan Xiaoliu Soup + TACE, f: Fuzheng Jiedu Formula + TACE, g: Yangzheng Xiaoji Capsule + TACE, h: Huai Er Granule + TACE, i: Anti-cancer Formula + TACE, j: Songyou Drink + TACE, k: Jianpi Huayu Formula + TACE, l: Weitiao Formula II + TACE, m: Compound Hongdoushan Capsule + TACE, n: Zhengan Huazheng Formula + TACE, o: Ganfu Formula + TACE, p: Compound Banmao Capsule + TACE, q: Fuzheng Quxie Anti-cancer Formula + TACE.

**FIGURE 9:**
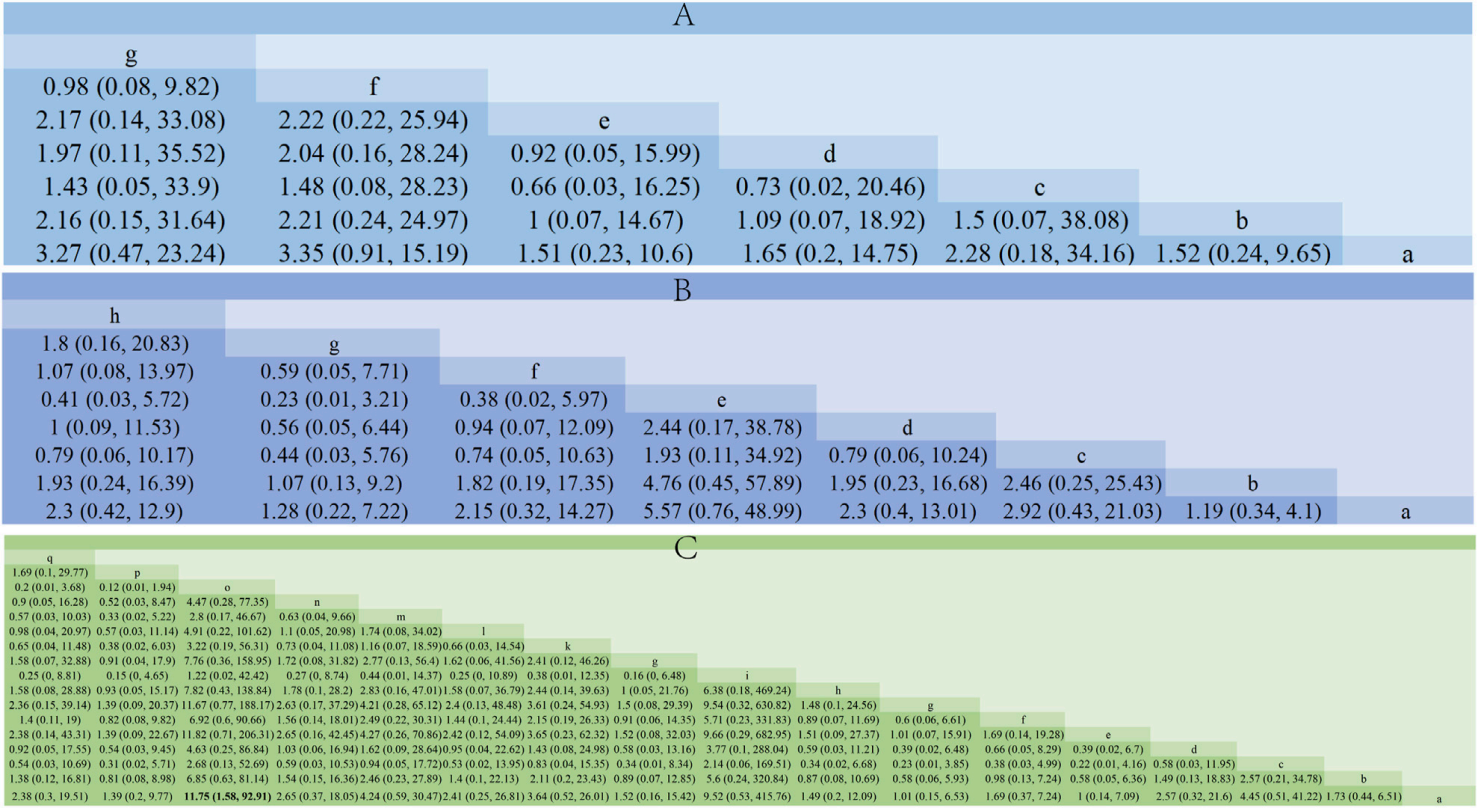
Comparison of network meta-analysis results. **(A)** Liver-soothing (Shugan) herbs. **(B)** Resolving blood stasis (Huayu) herbs. **(C)** Supporting vital qi (Fuzheng) herbs. Notes: in **(A)** a: TACE, b: Shanxian Granule + TACE, c: E Tao Soup + TACE, d: Weitiao Formula II + TACE, e: Xiaoyao Powder + TACE, f: Xiao Chaihu Soup + TACE, g: Chaihu Shugan Powder + TACE. in **(B)** a: TACE, b: Guben Yiliu Formula II + TACE, c: Yiqi Huoxue Soup + TACE, d: Jinlong Capsule + TACE, e: Anti-cancer Formula + TACE, f: Jianpi Xiaotan Sanjie Formula + TACE, g: Ge Xia Zhu Yu Soup + TACE, h: Huangqin Soup + TACE. in **(C)** a: TACE, b: Jianpi Liqi Principle + TACE, c: Xiao Zheng Fuzheng Soup + TACE, d: Fuzheng Anti-cancer Formula + TACE, e: Fuzheng Pinggan Xiaoliu Soup + TACE, f: Fuzheng Jiedu Formula + TACE, g: Yangzheng Xiaoji Capsule + TACE, h: Huai Er Granule + TACE, i: Anti-cancer Formula + TACE, j: Songyou Drink + TACE, k: Jianpi Huayu Formula + TACE, l: Weitiao Formula II + TACE, m: Compound Hongdoushan Capsule + TACE, n: Zhengan Huazheng Formula + TACE, o: Ganfu Formula + TACE, p: Compound Banmao Capsule + TACE, q: Fuzheng Quxie Anti-cancer Formula + TACE.

Additionally, we calculated the probabilities associated with each treatment plan’s ranking. Chaihu Shugan Powder in the liver-soothing (Shugan) group, Anti-Cancer Formula in the blood stasis-resolving (Huayu) group, and Ganfu Formula in the supporting vital qi (Fuzheng) group were most likely to be the most effective TCMs in their respective categories (Figure 8). The ranking table (Figure 9) shows that compared to TACE alone, all types of combined TCM and TACE treatment improved patients’ ORR, with vitality-supporting Ganfu Formula combined with TACE significantly improving ORR [OR = 11.75, 95% CI (1.58, 92.91)].

#### 3.4.6 Hub TCM mining

Analysis of TCM formulations identified the 10 most frequently used herbs in this study: “Baizhu,” “Huangqi,” “Fuling,” “Dangshen,” “Gancao,” “Ezhu,” “Chaihu,” “Baihuasheshecao,” “Baishao,” and “Chenpi” (Table 1). A novel formula combining these herbs was developed for subsequent NPA.

**TABLE 1:** Characteristics of ranking top 10 TCMs.

Pharmaceutical name	Chinese name	Counts	Frequency 1 (counts/total herb counts)	Frequency 2 (counts/study numbers)
Atractylodes Macrocephala Koidz	Baizhu	23	6.12%	58.97%
Hedysarum Multijugum Maxim	Huangqi	22	5.85%	56.41%
Poria Cocos (Schw.) Wolf	Fuling	18	4.79%	46.15%
Codonopsis Radix	Dangshen	16	4.26%	41.03%
licorice	Gancao	14	3.72%	35.90%
Curcumae Rhizoma	Ezhu	14	3.72%	35.90%
Radix Bupleuri	Chaihu	12	3.19%	30.77%
Hedyotis Diffusae Herba	Baihuasheshecao	11	2.93%	28.21%
Paeoniae Radix Alba	Baishao	10	2.66%	25.64%
Citrus Reticulata	Chenpi	9	2.39%	23.08%
Curcumae Radix	Yujin	8	2.13%	20.51%
Angelicae Sinensis Radix	Danggui	8	2.13%	20.51%
Scutellariae Barbatae Herba	Banzhilian	8	2.13%	20.51%

#### 3.4.7 Drug-HCC gene screening and drug-component-target construction

**FIGURE 10:**
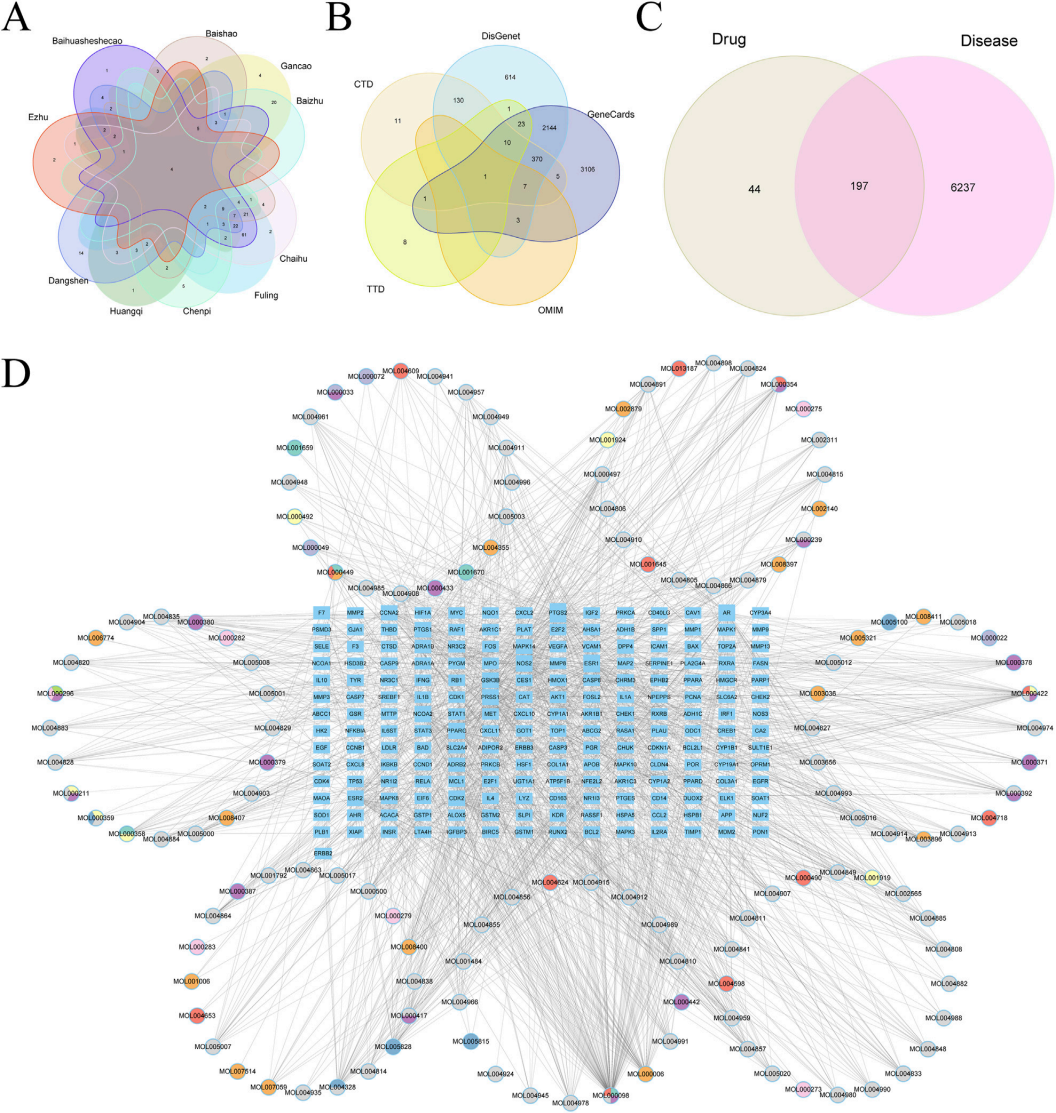
**(A)** Vene diagram of gene targets of ranking top 10 TCMs. **(B)** Vene diagram of multiple databases of disease targets for HCC. **(C)** Vene graph of TCM targets and HCC targets. **(D)** Ingredient-target-disease relationship network and PPI network diagram.

Target genes were identified for components from the 10 prominent TCMs, containing 139 compounds and 241 target genes, illustrated in a Venn diagram (Figure 10A). We retrieved 6,434 HCC-associated genes from five databases for analysis (Figure 10B). After intersecting the drug and disease genes, 197 intersection genes were obtained (Figure 10C). These were used alongside drug components to construct a network in Cytoscape, visualizing the interactions between drug compounds and target genes (Figure 10D).

#### 3.4.8 Core gene selection and functional enrichment analysis

**FIGURE 11:**
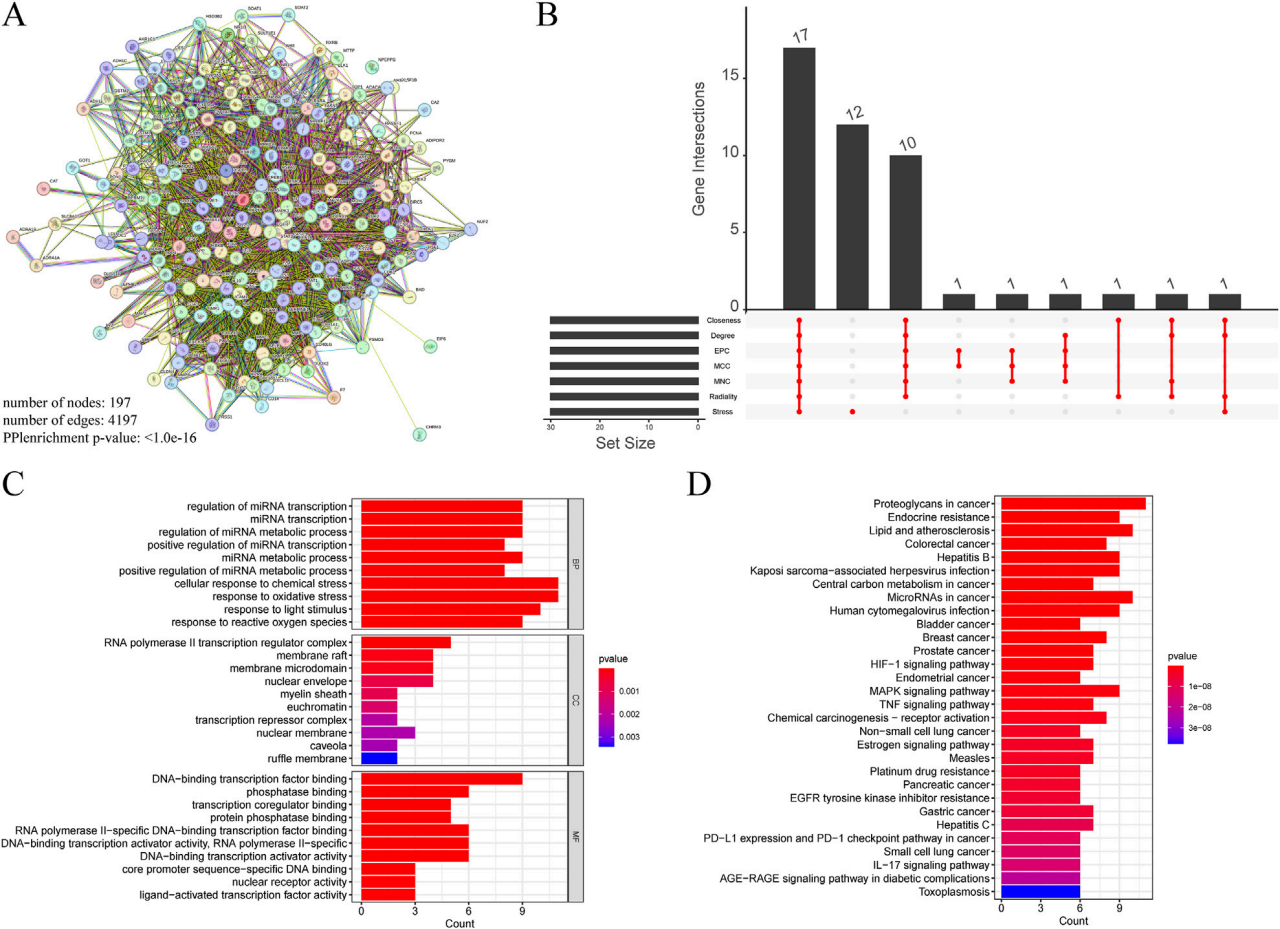
**(A)** PPI networks of cross genes between TCM targets and HCC targets. **(B)** The Venn diagram shows 17 core genes identified by seven algorithms. **(C)** GO analysis of core genes. **(D)** KEGG analysis of core genes.

A total of 197 intersecting genes were submitted to STRING for analysis (Figure 11A). Using seven algorithms in the cytoHubba plugin, we ranked genes according to score, with higher scores corresponding to higher ranks, and selected the top 30 genes for each algorithm (Supplementary Table S4). After examining the Venn diagram intersection, we identified 17 core genes that satisfied all seven algorithms, including AKT1, TP53, IL1β, STAT3, EGFR, PTGS2, CASP3, ESR1, HIF1A, MMP9, BCL2, MYC, PPARG, MAPK3, FOS, ERBB2, and IL10 (Figure 11B). GO enrichment analysis (Figure 11C) showed that in biological processes (BP), genes primarily regulate cellular response to chemical stress and response to oxidative stress; in cellular components (CC), the RNA polymerase II transcription regulator complex exhibited the highest enrichment. In the molecular function (MF) category, genes were predominantly associated with DNA-binding transcription factor binding. KEGG analysis indicated significant enrichment of proteoglycans in cancer, lipid and atherosclerosis in positively regulated pathways, and toxoplasmosis in negatively regulated pathways (Figure 11D).

#### 3.4.9 Molecular docking results

**FIGURE 12:**
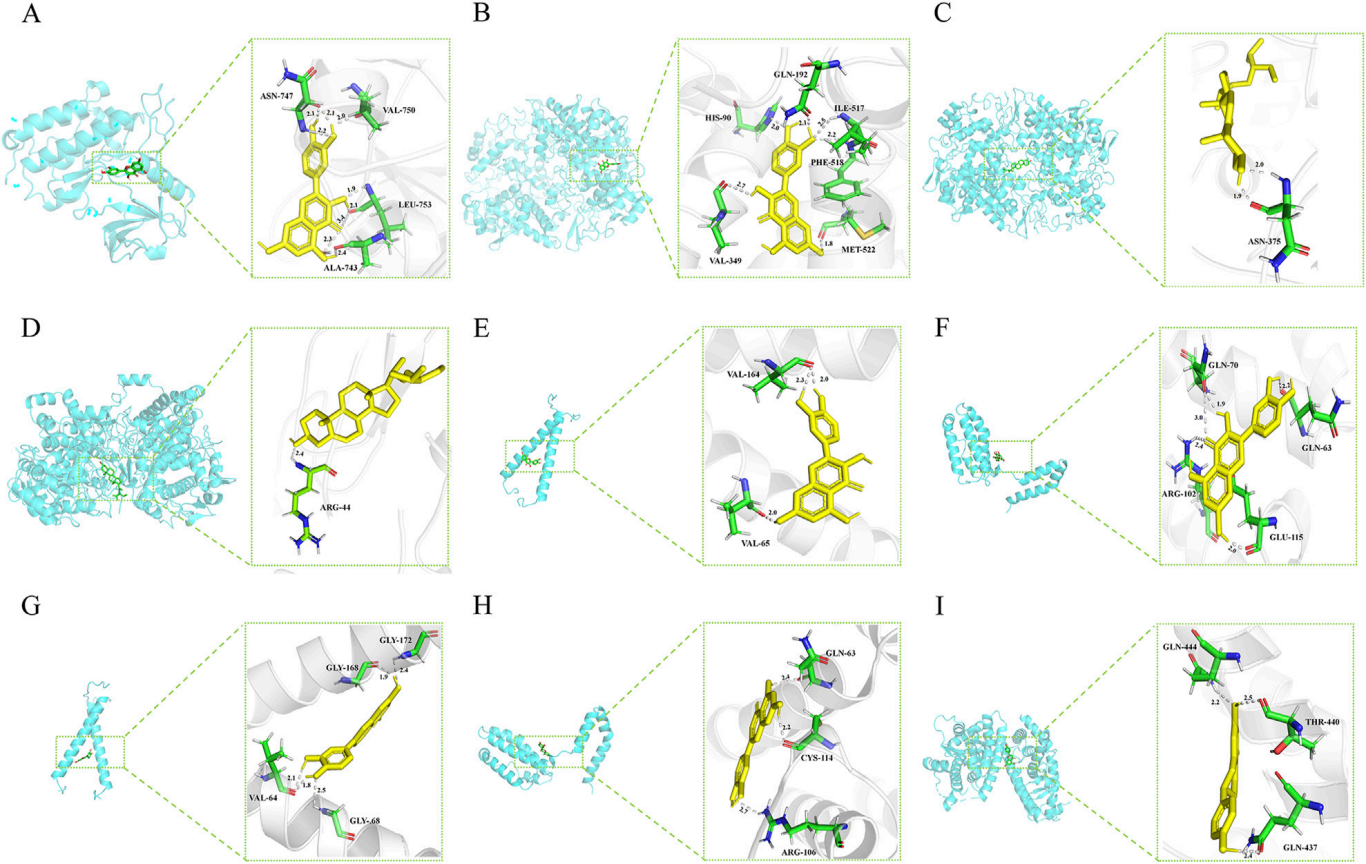
**(A)** EGFR (P00533) and quercetin. **(B)** PTGS2(P35354) and quercetin. **(C)** PTGS2 (P35354) and beta-sitosterol. **(D)** PTGS2 (P35354) and stigmasterol. **(E)** ERBB2 (P04626) and quercetin. **(F)** IL10 (P22301) and quercetin. **(G)** ERBB2 (P04626) and Luteolin. **(H)** IL10 (P22301) and Luteolin. **(I)** PPARG (P37231) and naringenin.

Molecular docking experiments were performed for each of the 17 selected core genes and their corresponding small-molecule ligands. Binding energies below −5 kJ/mol were considered indicative of binding stability, whereas energies below −7 kJ/mol suggested strong binding stability (Kitchen et al., 2004). Results indicated that ERBB2, IL10, and PPARG exhibited measurable binding abilities with their corresponding ligands, while EGFR and PTGS2 displayed stable ligand binding (Table 2). Protein identifiers were retrieved from the UniProt database for the genes: EGFR (P00533), PTGS2 (P35354), ERBB2 (P04626), IL10 (P22301), and PPARG (P37231). Quercetin formed hydrogen bonds with EGFR at ASN-747, VAL-750, LEU-753, and ALA-743 (Figure 12A). For PTGS2, quercetin formed hydrogen bonds at GLN-192, ILE-517, PHE-518, MET-522, VAL-349, and HIS-90 (Figure 12B), while beta-sitosterol and stigmasterol formed hydrogen bonds at ASN-375 and ARG-44, respectively (Figures 12C, D). Quercetin formed hydrogen bonds with ERBB2 at VAL-164 and VAL-65, and with IL10 at GLN-70, GLN-63, ARG-102, and GLU-115 (Figures 12E, F). Luteolin formed hydrogen bonds with ERBB2 at GLY-172, GLY-168, VAL-64, and GLY-68, and with IL-10 at GLN-63, CYS-114, and ARG-106 (Figures 12G, H). PPARG formed hydrogen bonds with naringenin at GLN-444, THR-440, and GLN-437 (Figure 12I).

**TABLE 2:** Lowest binding energy between active ingredient and key protein targets.

Name of Chinese medicine	Number	Active ingredient	Protein name	Lowest binding energy (kJ/mol)
BaiHuaSheSheCao, Chaihu, Gancao, Huangqi	MOL000098	quercetin	EGFR	−8.88
BaiHuaSheSheCao, Chaihu, Gancao, Huangqi	MOL000098	quercetin	PTGS2	−10.80
BaiHuaSheSheCao, Baishao	MOL000358	beta-sitosterol	PTGS2	−11.96
BaiHuaSheSheCao, Chaihu, Dangshen	MOL000449	Stigmasterol	PTGS2	−12.56
BaiHuaSheSheCao, Chaihu, Gancao, Huangqi	MOL000098	quercetin	ERBB2	−6.47
Dangshen	MOL000006	luteolin	ERBB2	−6.38
BaiHuaSheSheCao, Chaihu, Gancao, Huangqi	MOL000098	quercetin	IL10	−5.98
Dangshen	MOL000006	luteolin	IL10	−6.40
ChenPi, Gancao	MOL004328	naringenin	PPARG	−6.25

#### 3.4.10 Additional validation sets and *in vitro* experiments

**FIGURE 13:**
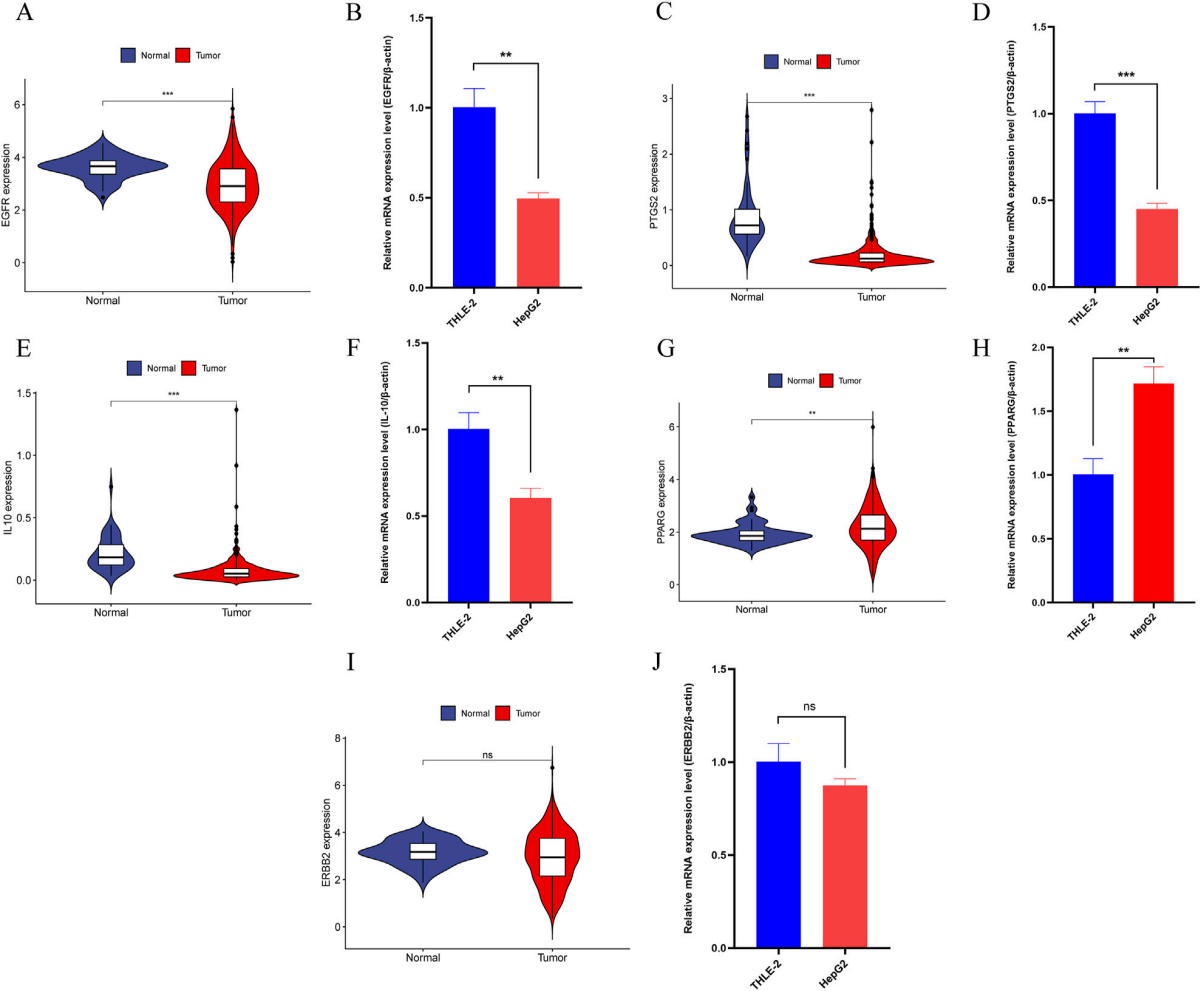
**(A)** The expression of EGFR in the TCGA-LIHC dataset. **(B)** qRT-PCR Results of EGFR in HCC cell lines. **(C)** The expression of PTGS2 in the TCGA-LIHC dataset. **(D)** qRT-PCR Results of PTGS2 in HCC cell lines. **(E)** The expression of IL10 in the TCGA-LIHC dataset. **(F)** qRT-PCR Results of IL10 in HCC cell lines. **(G)** The expression of PPARG in the TCGA-LIHC dataset. **(H)** qRT-PCR Results of PPARG in HCC cell lines. **(I)** The expression of ERBB2 in the TCGA-LIHC dataset. **(J)** qRT-PCR Results of ERBB2 in HCC cell lines. Note: “*”, “**”, and “***” indicate *p* < 0.05, *p* < 0.01, and *p* < 0.001, respectively. “ns” indicates *p* > 0.05.

In further analysis, we selected core genes with higher binding stability and validated their expression using the TCGA-LIHC dataset and qRT-PCR experiments. Results from both methods were consistent, showing that in HCC, the expression levels of EGFR, PTGS2, and IL10 were significantly downregulated (Figures 13A–F), whereas PPARG expression was notably upregulated (Figures 13G, H). However, no significant difference was detected in ERBB2 gene expression (Figures 13I, J).

## 4 Discussion

This study demonstrates that TCM combined with TACE treatment for HCC has significant clinical effects, providing strong evidence supporting this integrated treatment approach. Traditional Chinese Medicine attributes HCC pathogenesis to factors such as Qi stagnation, blood stasis, and accumulation of toxins, which purportedly weaken Zheng Qi and trap pathogenic Qi internally (Wang et al., 2015). TCM treatment for HCC emphasizes holistic adjustment, focusing on regulating liver Qi, strengthening the body’s foundation, and promoting blood circulation, tailored to patients through differential diagnosis (Liao et al., 2020). Our meta-analysis results reveal that TCM, when used alongside TACE, significantly enhances ORR, DCR, and OS, and reduces the incidence of AEs compared to TACE alone. These findings indicate that TCM may be effectively utilized as an effective adjunct to conventional cancer treatments. Notably, the Bayesian network meta-analysis particularly highlights the significant effects of supporting vital qi (Fuzheng) herbs like Ganfu Formula combined with TACE in improving ORR. Ganfu Formula contains ingredients like Hedyotis Diffusae Herba and Scutellariae Barbatae Herba, which are extensively utilized to suppress cancer cell proliferation and promote tumor cell apoptosis, demonstrating notable anti-tumor effects in various experimental models (Yang et al., 2021; Hou et al., 2024). Hedysarum Multijugum Maxim. and Codonopsis Radix enhance immune function and liver health, with Hedysarum Multijugum Maxim. also exhibiting antioxidant and anti-inflammatory effects, thereby reducing liver damage (Sheik et al., 2021). Curcumae Radix and Radix Bupleuri significantly alleviate liver Qi stagnation and blood stasis, helping reduce pain and anxiety in HCC patients and decrease tumor growth (Liu et al., 2022; Li et al., 2021). Pinellia and Licorice have expectorant, anti-inflammatory, and detoxifying effects, helping reduce AEs in HCC treatment (Bai et al., 2022; Aipire et al., 2017). These results provide additional evidence for the potential of TCM to modulate the tumor microenvironment, enhance immune responses, and mitigate TACE-related toxicity. However, due to the specific nature of TACE treatment, this study has limitations, including the absence of double-blinding and allocation concealment, leading to poor methodological quality. Additionally, the lack of sufficient literature prevents the evaluation of the impact of different categories of TCM on AEs in patients. Future high-quality RCTs should update meta-analysis conclusions to refine the data on AEs associated with TCM combined with TACE treatment for HCC. Additionally, this study does not investigate the *in vivo* metabolites of TCM or their specific biological activities, which limits a comprehensive understanding of TCM mechanisms, particularly regarding its potential effects on tumor microenvironment modulation and immune response enhancement. As TCM metabolites may exhibit pharmacological activities distinct from the original compounds, future studies will apply advanced analytical techniques, such as high-performance liquid chromatography-mass spectrometry, to systematically analyze these metabolites and their bioactivities. This will help elucidate the precise role of these metabolites in cancer treatment and provide a theoretical basis for optimizing the combined TCM and TACE strategy for HCC treatment.

NPA identified several key targets, including EGFR, PTGS2, ERBB2, IL10, PPARG, etc., that are intimately linked to the initiation and progression of HCC (Lin et al., 2024). BP analysis revealed that these genes primarily regulate cellular responses to chemical and oxidative stress. Consistent with existing literature, this suggests a strong link between HCC development and the body’s response to internal and external stimuli (Marra et al., 2011). TCM may boost the body’s stress resistance and inhibit tumor growth by modulating these genes’ expression. Interactions between RNA polymerase II and transcription factors regulate the expression of many tumor-related genes, crucial for the growth and differentiation of tumor cells (Martin, Hébert, and Tanny, 2020). CC analysis indicated significant enrichment of the RNA polymerase II transcription regulator complex, underscoring its role in HCC cell proliferation and metastasis. KEGG pathway analysis revealed that TCM may exert its anti-tumor effects by regulating various cancer-related signaling pathways, especially those involving proteoglycans and lipid metabolism. Proteoglycans are crucial in regulating cell proliferation, differentiation, and migration in HCC. Additionally, they influence the tumor microenvironment by modulating the extracellular matrix and cell signaling pathways (Wei et al., 2020). Additionally, lipid metabolism is closely linked to HCC development (Pope et al., 2019), with abnormal lipid metabolism promoting HCC cell growth via activation of the AKT/mTOR/SREBP1 pathway (Yin et al., 2017).

Quercetin suppresses cancer cell growth and triggers apoptosis by blocking EGFR-induced phosphorylation and downstream signaling pathways (Sharmila et al., 2014). Our docking analysis also indicates that quercetin forms hydrogen bonds at critical sites on the EGFR protein, suggesting that quercetin may regulate the proliferation and apoptosis of HCC cells by affecting EGFR signaling pathways. Additionally, studies show that quercetin and beta-sitosterol reduce cancer-related inflammation and tumor growth by inhibiting the expression and activity of PTGS2 (Manukyan, 2020; Salamatullah et al., 2021), supporting the relevance of our docking results where quercetin binds to PTGS2. Stigmasterol mitigates inflammation through the suppression of the NF-κB signaling pathway (Ahmad Khan et al., 2020) and impedes cancer cell proliferation and survival by targeting the Akt/mTOR and JAK/STAT pathways (Bakrim et al., 2022). Although existing studies have not directly proven that stigmasterol inhibits PTGS2 activity by binding to it, our molecular docking studies observed that stigmasterol can form stable hydrogen bonds with PTGS2, potentially reducing its activity and thereby inhibiting inflammation-related enzymatic reactions associated with PTGS2. The TCGA-LIHC and qRT-PCR results both indicate that EGFR and PTGS2 expression is significantly downregulated in HCC, providing preliminary support for the reliability of the molecular docking results in this study. Although these findings offer strong initial evidence and new insights for drug design and development, further experimental validation is required in the future.

## 5 Conclusion

In summary, TCM combined with TACE treatment shows more significant clinical efficacy and safety compared to TACE alone. NPA and molecular docking have uncovered multiple potential targets and their interactions pertinent to HCC treatment, offering insights into the disease mechanisms and references for further basic research and new drug development.

## Data Availability

The original contributions presented in the study are included in the article/Supplementary Material, further inquiries can be directed to the corresponding author.
