# Supplementary material for: Efficacy and safety of TACE combined with traditional Chinese medicine versus TACE alone in hepatocellular carcinoma: bayesian network meta-analysis and pharmacological mechanisms study
Source: Front Pharmacol. 2024 Dec 6;15:1495343. doi: 10.3389/fphar.2024.1495343 (PMC11662279; doi:10.3389/fphar.2024.1495343)
Supplement: Supplementary file 1 [file DataSheet1.docx]

**Supplementary materials**

| **Supplementary Table S1.** Retrieval strategies | | |
| --- | --- | --- |
| **Database** | **Number** | **Search terms** |
| **PubMed** | #1 | ("Medicine, Chinese Traditional"[MeSH] OR "Drugs, Chinese Herbal"[MeSH] OR "Phytotherapy"[MeSH] OR "Traditional Chinese Medicine"[Title/Abstract] OR "TCM"[Title/Abstract] OR "Chinese Herbal Medicine"[Title/Abstract] OR "Chinese Medicine"[Title/Abstract] OR "Herbal Medicine"[Title/Abstract] OR "Phytotherapy"[Title/Abstract] OR "Chinese herbs"[Title/Abstract]) |
|  | #2 | ("Carcinoma, Hepatocellular"[MeSH] OR "Liver Neoplasms"[MeSH] OR "Hepatocellular Carcinoma"[Title/Abstract] OR "Liver Cancer"[Title/Abstract] OR "Hepatic Cancer"[Title/Abstract] OR "HCC"[Title/Abstract]) |
|  | #3 | ("randomized controlled trials as topic" OR "controlled clinical trial*" OR "randomized*" OR "placebo" OR "clinical trial*" OR "controlled trial*") |
|  | #4 | #1 AND #2 AND #3 |
| **Embase** | #1 | 'Chinese herbal medicine' OR 'Chinese traditional medicine' OR 'medicine, Chinese traditional' OR 'traditional Chinese medicine' OR 'Chinese medicine' |
|  | #2 | 'carcinoma in the liver' OR 'carcinoma of the liver' OR 'carcinoma, hepatic cell' OR 'carcinoma, hepatocellular' OR 'carcinoma, liver' OR 'carcinoma, liver cell' OR 'hepatic carcinoma' OR 'hepatic cell carcinoma' OR 'hepato-carcinoma' OR 'hepato-cellular carcinoma' OR 'hepatocarcinoma' OR 'hepatocellular carcinoma' OR 'hepatocellular carcinomata' OR 'hepatocyte carcinoma' OR 'hepatocytic carcinoma' OR 'hepatoma' OR 'hepatomata' OR 'hepatomatous' OR 'liver carcinoma' OR 'liver carcinoma rupture' OR 'malignant hepatoma' OR 'primary liver carcinoma' OR 'liver cell carcinoma' |
|  | #3 | 'pragmatic clinical trials as topic' OR 'randomized controlled trials' OR 'randomized controlled trials as topic' OR 'randomized controlled trial (topic)' |
|  | #4 | #1 AND #2 AND #3 |
| **Web of Science** | #1 | ((TS=(randomized controlled trials OR controlled clinical trial OR randomized OR placebo OR clinical trial OR controlled trial)) AND TS=(Traditional Chinese Medicine OR TCM OR Chinese Herbal Medicine OR Chinese Medicine OR Herbal Medicine OR Phytotherapy OR Chinese herbs)) AND TS=(Carcinoma, Hepatocellular OR Liver Neoplasms OR Hepatocellular Carcinoma OR Liver Cancer OR Hepatic Cancer OR HCC) |
| **Cochrane Library** | #1 | MeSH descriptor: [Liver Neoplasms] explode all trees |
|  | #2 | (Cancers, Hepatic):ti,ab,kw OR (Liver Cancer):ti,ab,kw OR (Cancer, Liver):ti,ab,kw OR (Cancer of Liver):ti,ab,kw OR (Hepatocellular Cancers):ti,ab,kw OR (Hepatocellular Cancer):ti,ab,kw OR (Cancers, Hepatocellular):ti,ab,kw OR (Cancer, Hepatocellular):ti,ab,kw OR (Hepatic Cancers):ti,ab,kw OR (Liver Cancers):ti,ab,kw OR (Cancer of the Liver):ti,ab,kw OR (Cancers, Liver):ti,ab,kw OR (Hepatic Cancer):ti,ab,kw OR (Cancer, Hepatic):ti,ab,kw OR (Neoplasm, Liver):ti,ab,kw OR (Neoplasms, Hepatic):ti,ab,kw OR (Hepatic Neoplasms):ti,ab,kw OR (Neoplasms, Liver):ti,ab,kw OR (Hepatic Neoplasm):ti,ab,kw OR (Liver Neoplasm):ti,ab,kw OR |
|  | #3 | #1 OR #2 |
|  | #4 | MeSH descriptor: [Medicine, Chinese Traditional] explode all trees |
|  | #5 | (Hsueh, Chung I):ti,ab,kw OR (Traditional Chinese Medicine):ti,ab,kw OR (Zhong Yi Xue):ti,ab,kw OR (Traditional Medicine, Chinese):ti,ab,kw OR (Chung I Hsueh):ti,ab,kw OR (Chinese Traditional Medicine):ti,ab,kw OR (Tongue Diagnoses, Traditional):ti,ab,kw OR (Traditional Tongue Assessments):ti,ab,kw OR (Traditional Tongue Diagnoses):ti,ab,kw OR (Tongue Assessment, Traditional):ti,ab,kw OR (Traditional Tongue Assessment):ti,ab,kw OR (Tongue Diagnosis, Traditional):ti,ab,kw OR (Traditional Tongue Diagnosis):ti,ab,kw |
|  | #6 | #4 OR #5 |
|  | #7 | #3 AND #6 |
| **CNKI** | #1 | HCC OR (Hepatocellular carcinoma) OR (hepatoma) OR (liver Neoplasms) OR (liver cancer) |
|  | #2 | Chinese Traditional Medicine OR (Chinese Herbal Medicine) |
|  | #3 | randomized controlled trials OR (randomized clinical trial) OR (randomized experiment) OR (RCT) |
|  | #4 | #1 AND #2 AND #3 |
| **WanFang database** | #1 | HCC OR (Hepatocellular carcinoma) OR (hepatoma) OR (liver Neoplasms) OR (liver cancer) |
|  | #2 | Chinese Traditional Medicine OR (Chinese Herbal Medicine) |
|  | #3 | randomized controlled trials OR (randomized clinical trial) OR (randomized experiment) OR (RCT) |
|  | #4 | #1 AND #2 AND #3 |
| **VIP** | #1 | HCC OR (Hepatocellular carcinoma) OR (hepatoma) OR (liver Neoplasms) OR (liver cancer) |
|  | #2 | Chinese Traditional Medicine OR (Chinese Herbal Medicine) |
|  | #3 | randomized controlled trials OR (randomized clinical trial) OR (randomized experiment) OR (RCT) |
|  | #4 | #1 AND #2 AND #3 |
|  | #1 | HCC OR (Hepatocellular carcinoma) OR (hepatoma) OR (liver Neoplasms) OR (liver cancer) |
| **CBM** | #2 | Chinese Traditional Medicine OR (Chinese Herbal Medicine) |
|  | #3 | randomized controlled trials OR (randomized clinical trial) OR (randomized experiment) OR (RCT) |
|  | #4 | #1 AND #2 AND #3 |

**Supplementary Table S2.** Primers sequences used in this study

| Name | forward | reverse |
| --- | --- | --- |
| EGFR | TTGCCGCAAAGTGTGTAACG | GTCACCCCTAAATGCCACCG |
| PTGS2 | GGAAGCACTCTATGGTGACATC | CCAGTAGGCAGGAGAACATATAAC |
| ERBB2 | CCTGCTGAACTGGTGTATGC | TGGTACTCTGTCTCGTCAATGT |
| IL10 | AGCTGAGAACCAAGACCCAGA | ACTCATGGCTTTGTAGATGCCTT |
| PPARG | GACATTCAAGACAACCTGCTACA | CGTGTTCCGTGACAATCTGT |
| β-actin | GAGAAAATCTGGCACCACACC | GGATAGCACAGCCTGGATAGCAA |

**Supplementary Table S3.** Principal characteristics of all included studies in meta-analysis.

|  |  | Sample size(EG/CG) | Intervention | | sex（male/female) | | Mean age or age range | |  |
| --- | --- | --- | --- | --- | --- | --- | --- | --- | --- |
| ID | study |  | EG | CG | EG | CG | EG | CG | Outcomes |
| 1 | Zhou X.Y. 2002 | 228(118/110) | Jianpi Liqi Principle + TACE | TACE | 98/20 | 96/14 | 29-70 | 28-72 | DCR,ORR,OS |
| 2 | Shao Z.X. 2001 | 60(30/30) | Liver Cancer Formula I + TACE | TACE | 28/2 | 26/4 | 48.57±12.96 | 51.30±11.∙54 | OS |
| 3 | Ren H.P. 2004 | 172(104/68) | Jianpi Liqi Method + TACE | TACE | 94/10 | 60/8 | 51.2 | 53.9 | DCR,ORR,OS,AEs |
| 4 | Cao G.W. 2005 | 100(50/50) | Ganfu Kang Capsule + TACE | TACE | NA | NA | NA | NA | OS |
| 5 | Zhang C.Q. 2005 | 224(116/108) | Jinlong Capsule + TACE | TACE | 98/18 | 89/19 | 52.1±9.7 | 50.4±8.5 | OS |
| 6 | Zhang Y.M. 2005 | 92(50/42) | Shanxian Granule + TACE | TACE | 29/21 | 27/15 | NA | NA | DCR,ORR |
| 7 | Lin Y.H. 2005 | 50(25/25) | Jianpi Liqi Formula + TACE | TACE | NA | NA | NA | NA | OS |
| 8 | Yuan J.B. 2005 | 45(24/21) | Xiao Zheng Fuzheng Soup + TACE | TACE | 18/6 | 16/5 | 39-68 | 41-65 | DCR,ORR,  AEs |
| 9 | Qu Y.Y. 2006 | 50(28/22) | E Tao Soup + TACE | TACE | 23/5 | 19/3 | 25-65 | 38-79 | DCR,ORR,OS |
| 10 | Wen H.Y. 2006 | 62(32/30) | Fuzheng Anti-cancer Formula + TACE | TACE | 20/12 | 17/13 | 28-72 | 29-68 | DCR,ORR |
| 11 | Yu J. 2006 | 62(32/30) | Guben Yiliu Formula II + TACE | TACE | 26/6 | 25/5 | NA | NA | DCR,ORR,OS,AEs |
| 12 | Liu H.M. 2007 | 66(36/30) | Jianpi Jiedu Huazheng Soup + TACE | TACE | 20/16 | 20/10 | 35-65 | 35-65 | OS |
| 13 | Wang Z.F. 2007 | 95(60/35) | Fuzheng Pinggan Xiaoliu Soup + TACE | TACE | 41/19 | 26/9 | 28-74 | 29-68 | DCR,ORR,OS |
| 14 | Zhang Q. 2007 | 112(58/54) | Guben Yiliu Formula II + TACE | TACE | 38/20 | 35/19 | 56.5±8.5 | 58.0±7.0 | DCR,ORR,OS,AEs |
| 15 | Zhong R. 2007 | 50(26/24) | Yiqi Huoxue Soup + TACE | TACE | 20/6 | 19/5 | 43-65 | 40-65 | DCR,ORR |
| 16 | Wu G.L. 2010 | 98(45/53) | Jinlong Capsule + TACE | TACE | 39/6 | 44/9 | 40-70 | 36-70 | DCR,ORR |
| 17 | Zhou X.Z. 2010 | 64(32/32) | Anti-cancer Formula + TACE | TACE | NA | NA | NA | NA | DCR,ORR,OS |
| 18 | Han K.Q. 2013 | 93(47/46) | Fuzheng Jiedu Formula + TACE | TACE | 39/8 | 38/8 | 25-75 | 22-76 | DCR,ORR,OS |
| 19 | Liu X. 2013 | 64(32/32) | Weitiao Formula II + TACE | TACE | 27/5 | 26/6 | 52.2±9.74 | 51.7±10.30 | DCR,ORR |
| 20 | Song D.G. 2013 | 457(232/225) | Yangzheng Xiaoji Capsule + TACE | TACE | 124/108 | 122/103 | NA | NA | DCR,ORR,  AEs |
| 21 | Zhao G.S. 2013 | 62(31/31) | Huai Er Granule + TACE | TACE | 27/4 | 29/2 | 64.9 | 62.7 | DCR,ORR,OS,AES |
| 22 | Yin T.L. 2013 | 21(11/10) | Anti-cancer Formula + TACE | TACE | NA | NA | NA | NA | DCR,ORR |
| 23 | Li B. 2014 | 62(32/30) | Jianpi Xiaotan Sanjie Formula + TACE | TACE | 23/9 | 19/11 | 34-65 | 31-63 | ORR |
| 24 | Zheng S.S. 2014 | 44(23/21) | Songyou Drink + TACE | TACE | 21/2 | 19/2 | 55.52±10.61 | 52.62±9.95 | DCR,ORR,OS |
| 25 | Li H.T. 2015 | 204(102/102) | Jianpi Huayu Formula + TACE | TACE | 52/50 | 53/49 | 57.3±1.6 | 57.5±1.7 | ORR,OS,AES |
| 26 | Tang Z.J. 2017 | 60(30/30) | Weitiao Formula II + TACE | TACE | 24/6 | 25/5 | 66.83±10.25 | 63.20±9.30 | DCR,ORR,  AEs |
| 27 | Ding H.B. 2018 | 122(61/61) | Compound Hongdoushan Capsule + TACE | TACE | 40/21 | 41/20 | 57.02±11.54 | 57.03±10.95 | DCR,ORR |
| 28 | Wan X.Y. 2018 | 110(55/55) | Zhengan Huazheng Formula + TACE | TACE | 31/24 | 33/22 | 53.68±10.37 | 53.52±10.30 | DCR,ORR |
| 29 | Yang H. 2018 | 60(30/30) | Xiaoyao Powder + TACE | TACE | 23/7 | 24/6 | 51.3±9.29 | 53.72±10.80 | DCR,ORR,  AEs |
| 30 | Huang R.Y. 2019 | 100(50/50) | Ge Xia Zhu Yu Soup + TACE | TACE | 34/16 | 32/18 | 50.9±7.4 | 50.7±7.5 | DCR,ORR |
| 31 | Li Y. 2019 | 112(56/56) | Huangqin Soup + TACE | TACE | 41/15 | 43/13 | 46.15±6.27 | 47.24±4.68 | DCR,ORR |
| 32 | Qiao J.J 2019 | 120(60/60) | Ganfu Formula + TACE | TACE | 29/31 | 29/31 | 61.56±2.11 | 62.86±1.82 | ORR,AEs |
| 33 | Tian M.M. 2019 | 114(57/57) | Xiao Chaihu Soup + TACE | TACE | 39/18 | 38/19 | 62±14.79 | 61±13.19 | DCR,ORR |
| 34 | Qiao X.T. 2019 | 102(51/51) | Fuyuan Huoxue Soup Hehuaji Pill + TACE | TACE | 34/17 | 36/15 | 46.61±5.27 | 47.13±5.08 | OS,AEs |
| 35 | Hong G.H. 2020 | 100(50/50) | Compound Banmao Capsule + TACE | TACE | 27/23 | 26/24 | 53.78±6.45 | 53.80±6.23 | DCR,ORR |
| 36 | Wang X.D. 2020 | 50(25/25) | Fuzheng Quxie Anti-cancer Formula + TACE | TACE | 23/2 | 20/5 | 55.52±12.58 | 56.52±13.40 | DCR,ORR |
| 37 | Meng F.R. 2021 | 120(60/60) | Modified Xiao Chaihu Soup + TACE | TACE | 41/19 | 39/21 | 53.28±4.16 | 52.43±4.76 | DCR,ORR |
| 38 | Yan T.F. 2021 | 60(30/30) | Chaihu Shugan Powder + TACE | TACE | 17/13 | 18/12 | 57.13±8.26 | 57.27±9.63 | DCR,ORR,OS,AEs |
| 39 | Wu T. 2022 | 72(36/36) | Fuzheng Jiedu Xiaoji Formula + TACE | TACE | 26/10 | 30/6 | 44.5±3.2 | 43.9±3.3 | DCR,ORR |

EG: Experiment group; CG: Control group; ORR: Overall response rate, was defined as the proportion of patients with complete response (CR) + partial response (PR) after treatment to the total number of patients; DCR: Disease control rate, was defined as the percentage of patients who achieved response (PR + CR) + stable disease (SD) after treatment to the total number of patients; OS: Overall survival; AEs: Adverse events.

**Supplementary Table S4.** Top 30 genes based on cytoHubba

| Closeness | Degree | EPC | MCC | MNC | Radiality | Stress |
| --- | --- | --- | --- | --- | --- | --- |
| AKT1 | AKT1 | MMP9 | MMP9 | AKT1 | AKT1 | AKT1 |
| TP53 | TP53 | AKT1 | AKT1 | TP53 | TP53 | TP53 |
| IL1β | IL1β | TP53 | TP53 | IL1β | IL1β | PTGS2 |
| STAT3 | STAT3 | EGFR | EGFR | STAT3 | STAT3 | IL1β |
| EGFR | EGFR | HIF1A | HIF1A | EGFR | EGFR | ESR1 |
| PTGS2 | CASP3 | STAT3 | STAT3 | CASP3 | PTGS2 | EGFR |
| CASP3 | PTGS2 | PTGS2 | PTGS2 | PTGS2 | ESR1 | MAPK3 |
| ESR1 | ESR1 | BCL2 | BCL2 | ESR1 | CASP3 | PPARG |
| HIF1A | HIF1A | MYC | MYC | MMP9 | HIF1A | MMP9 |
| MMP9 | MMP9 | IL1β | IL1β | HIF1A | MMP9 | HIF1A |
| BCL2 | BCL2 | CASP3 | CASP3 | BCL2 | BCL2 | STAT3 |
| MYC | MYC | MAPK3 | MAPK3 | MYC | MYC | MYC |
| PPARG | PPARG | EGF | EGF | PPARG | PPARG | CASP3 |
| MAPK3 | MAPK3 | ESR1 | ESR1 | MAPK3 | MAPK3 | HMGCR |
| FOS | FOS | PPARG | PPARG | FOS | FOS | BCL2 |
| CCND1 | CCND1 | CCND1 | CCND1 | CCND1 | CCND1 | NFE2L2 |
| EGF | EGF | FOS | FOS | EGF | ERBB2 | FOS |
| ERBB2 | ERBB2 | NFKBIA | NFKBIA | ERBB2 | EGF | HMOX1 |
| CCL2 | CCL2 | ERBB2 | ERBB2 | CCL2 | CCL2 | MAOA |
| IFNG | IFNG | CCL2 | CCL2 | IFNG | GSK3B | APOB |
| GSK3B | GSK3B | CXCL8 | CXCL8 | GSK3B | IFNG | SLC6A2 |
| IL10 | IL10 | MMP2 | MMP2 | IL10 | IL10 | CYP3A4 |
| CXCL8 | CXCL8 | STAT1 | STAT1 | CXCL8 | CXCL8 | PPARA |
| MMP2 | MMP2 | IL10 | IL10 | MMP2 | ICAM1 | APP |
| ICAM1 | ICAM1 | ICAM1 | ICAM1 | ICAM1 | HMOX1 | GSTP1 |
| IL1A | IL1A | IFNG | IFNG | IL1A | MMP2 | HSPA5 |
| NFKBIA | NFKBIA | GSK3B | GSK3B | NFKBIA | NFKBIA | IL10 |
| HMOX1 | STAT1 | RELA | RELA | STAT1 | CREB1 | CASP9 |
| CREB1 | BCL2L1 | BCL2L1 | BCL2L1 | BCL2L1 | IL1A | ERBB2 |

EPC: Edge Percolated Component; MCC: Maximal Clique Centrality; MNC: Maximum Neighborhood Componen.


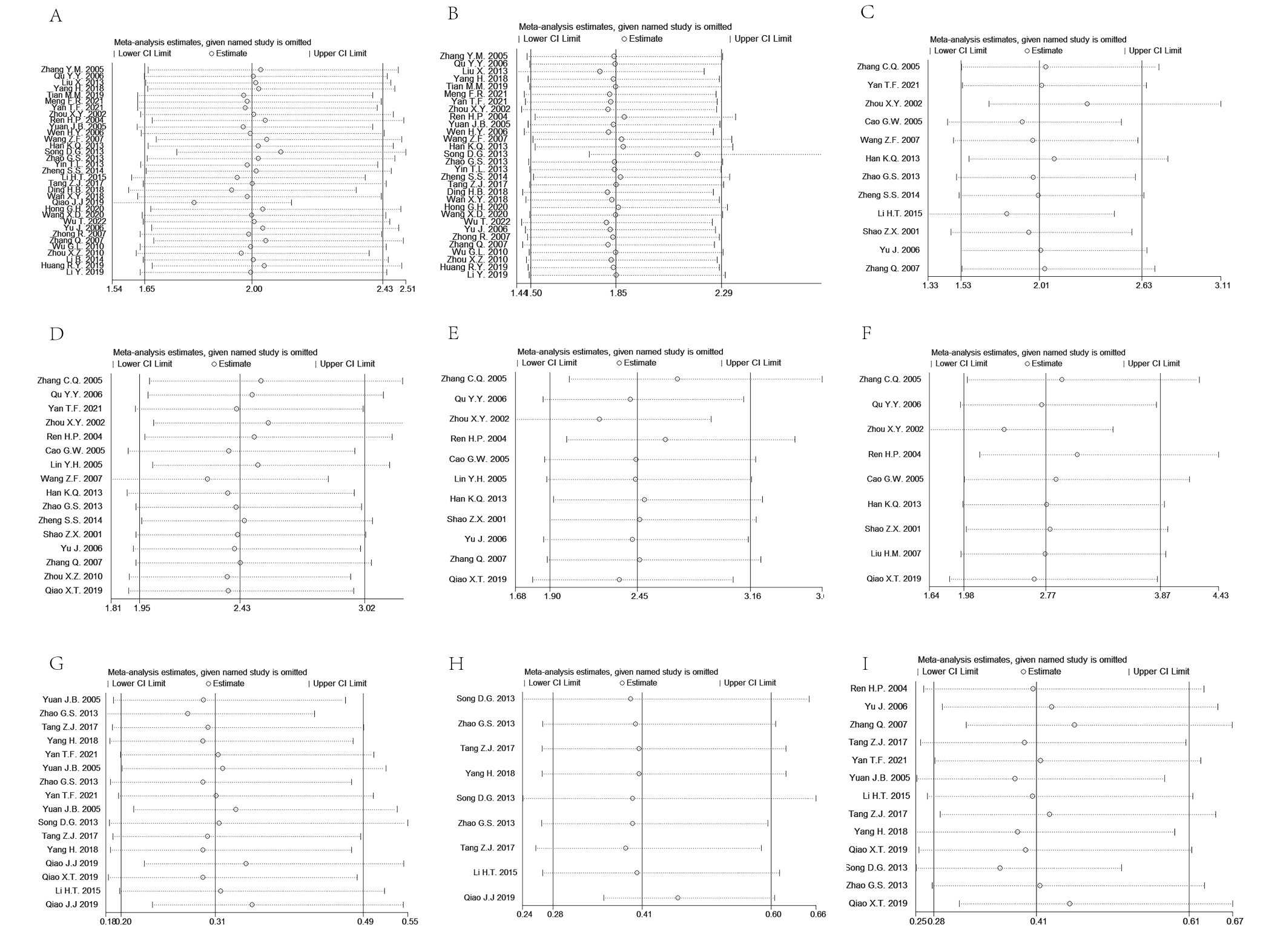


**Supplementary Figure S1.** Sensitivity analysis. **(A)** ORR. **(B)** DCR. **(C)** half-year OS. **(D)** one-year OS. **(E)** two-year OS. **(F)** three-year OS. **(G)** Digestive system complications. **(H)** Hematologic system complications. **(I)** Other complications.


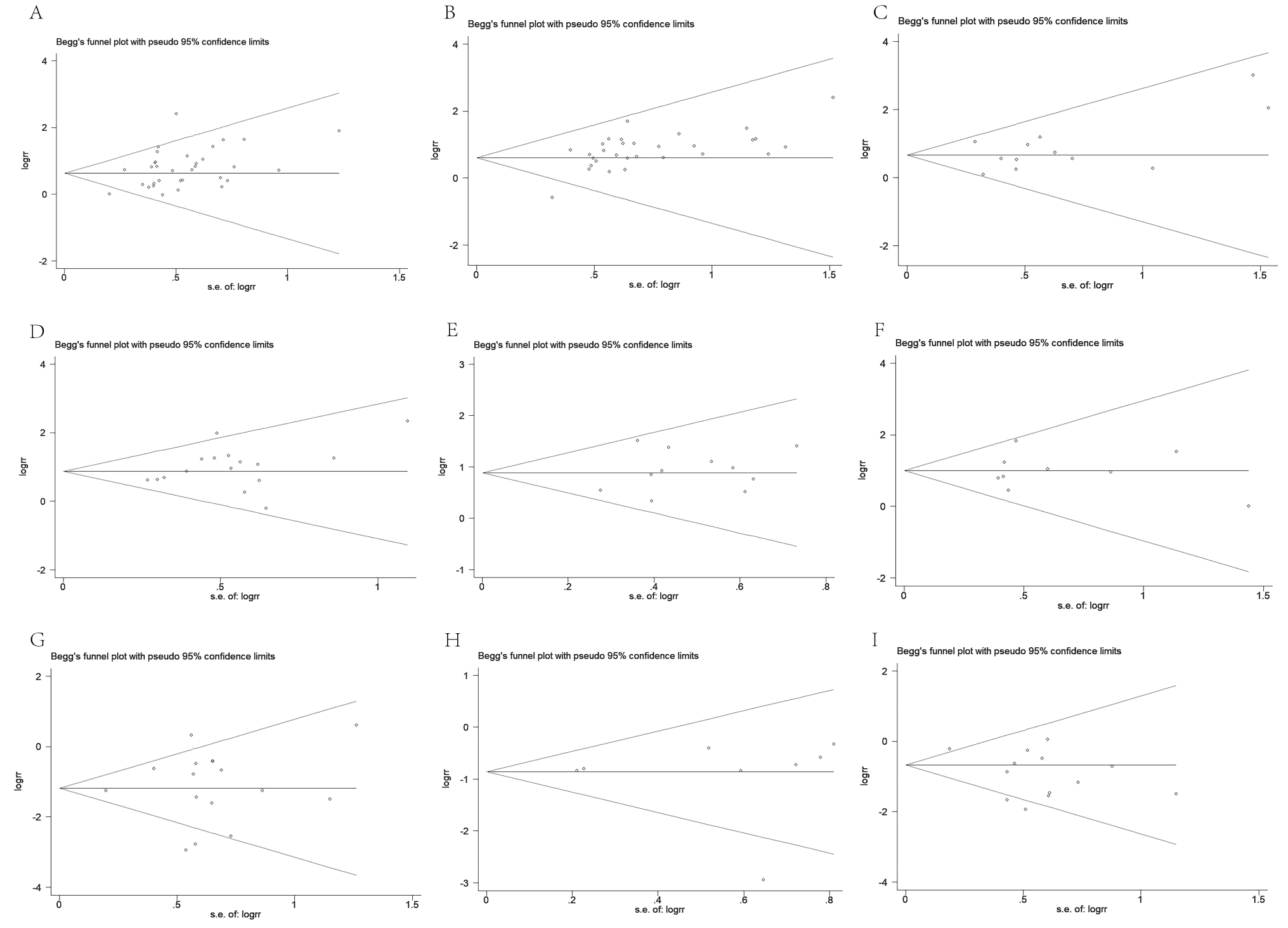


**Supplementary Figure S2.** Publication bias. **(A)** ORR. **(B)** DCR. **(C)** half-year OS. **(D)** one-year OS. **(E)** two-year OS. **(F)** three-year OS. **(G)** Digestive system complications. **(H)** Hematologic system complications. **(I)** Other complications.


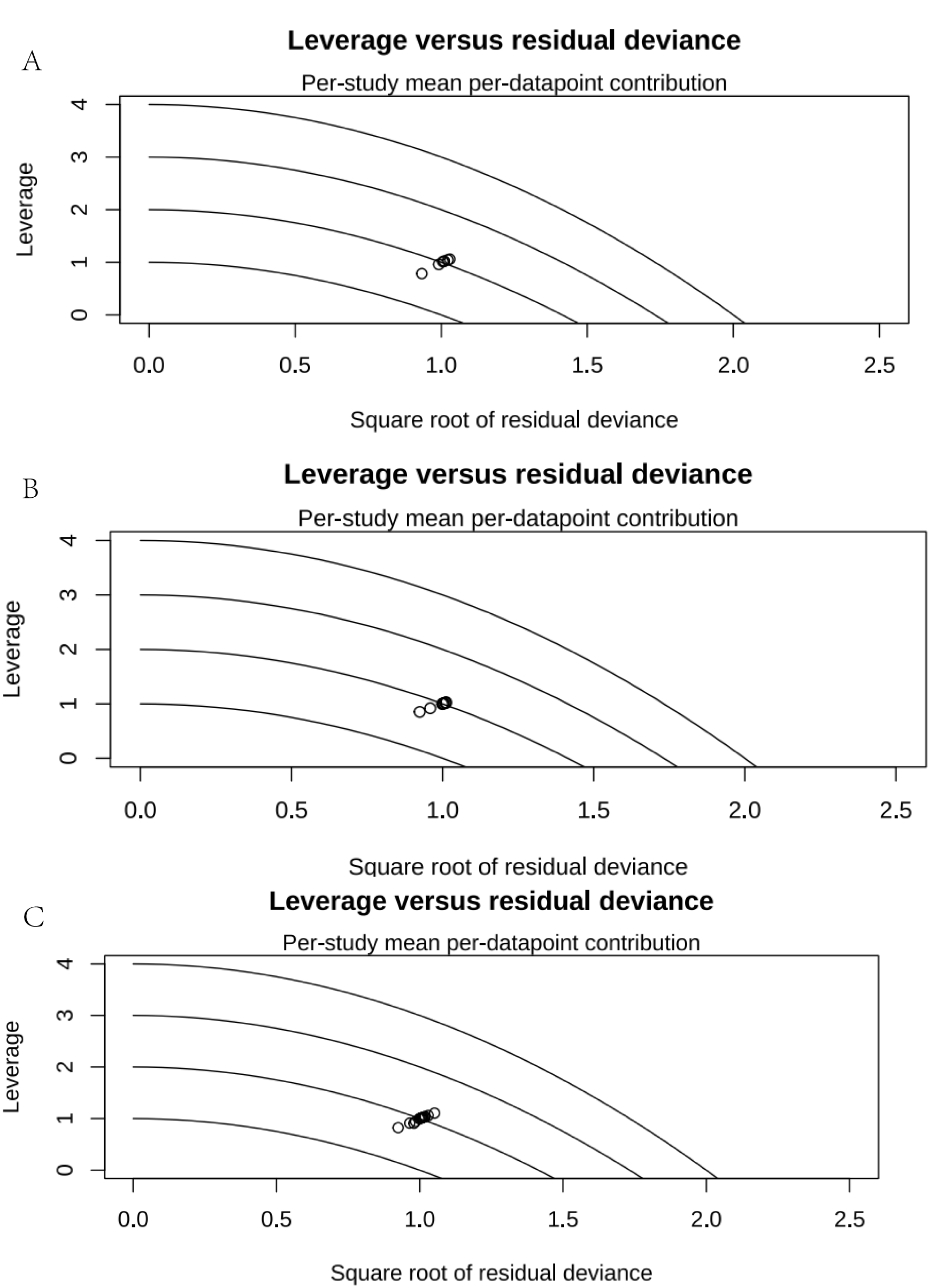


**Supplementary Figure S3** Leverage plot. **(A)**Liver-soothing (Shugan) herbs. **(B)** Resolving blood stasis (Huayu) herbs . **(C)** Supporting vital qi (Fuzheng) herbs.
